# Development and Validation of a Consumer-Oriented Sensory Evaluation Scale for Pale Lager Beer

**DOI:** 10.3390/foods14162834

**Published:** 2025-08-15

**Authors:** Yiyuan Chen, Ruiyang Yin, Liyun Guo, Dongrui Zhao, Baoguo Sun

**Affiliations:** 1 China Food Flavor and Nutrition Health Innovation Center, Beijing Technology and Business University, Beijing 100048, China; chenyiyuan_1112@163.com (Y.C.); sunbg@btbu.edu.cn (B.S.); 2 Beijing Key Laboratory of Beer Brewing Technology, Technology Center of Beijing Yanjing Beer Co., Ltd., Beijing 101300, China; ruiyang_yin@163.com (R.Y.); yanjing6089@163.com (L.G.); 3 Beijing Laboratory of Food Quality and Safety, Beijing Technology and Business University, Beijing 100048, China; 4 Key Laboratory of Brewing Molecular Engineering of China Light Industry, Beijing Technology and Business University, Beijing 100048, China

**Keywords:** pale lager, consumer perspective, sensory priority quantification, purchase decision, sensory evaluation scale, weighting methods, consumer behavior

## Abstract

Pale lager dominates global beer markets. However, rising living standards and changing consumer expectations have reshaped sensory preferences, highlighting the importance of understanding consumers’ true sensory priorities. In this study, a twenty-eight-item questionnaire, refined through multiple rounds of optimization, was distributed across China and yielded 1837 valid responses. Spearman correlation analysis and partial least-squares regressions showed that educational background and spending willingness exerted the strongest independent effects on sensory priorities. A hybrid analytic hierarchy process–entropy weight method–Delphi procedure was then applied to quantify sensory attribute importance. Results indicated that drinking sensation (30.92%) emerged as the leading driver of pale lager choice, followed by taste (26.60%), aroma (24.77%), and appearance (17.71%), confirming a flavor-led and experience-oriented preference structure. Weighting patterns differed across drinking-frequency cohorts: consumers moved from reliance on overall mouthfeel, through heightened sensitivity to negative attributes, to an eventual focus on subtle hedonic details. Based on these findings, a new sensory evaluation scale was developed and validated against consumer preference rankings, showing significantly stronger alignment with consumer preferences (ρ = 0.800; τ = 0.667) than the traditional scale. The findings supply actionable metrics and decision tools for breweries, supporting applications in product development, quality monitoring, and targeted marketing.

## 1. Introduction

Beer is the most widely consumed alcoholic beverage worldwide and occupies an irreplaceable position in the global drinks market [1]. It also contains soluble fibers; essential minerals such as calcium, iron, magnesium, phosphorus, potassium, zinc, manganese, and selenium; and a variety of vitamins, particularly from the B, A, D, and E groups. Scientific studies have shown that, due to the content of desired compounds, moderate beer consumption may contribute to certain health-promoting effects and physiological benefits [2]. Among the many beer styles, pale lager, noted for its brilliance, low alcohol strength, refreshing palate, and broad drinkability, became the most widely traded and largest-volume category internationally. In China, pale lager largely shaped the public conception of “beer,” and it routinely appeared at family dinners, social occasions, business banquets, and festive celebrations [3]. Over recent decades, the rising income levels and the ongoing upgrade of household consumption shifted beverage demand from basic hydration toward quality expression and personal identity. Within the mainstream market dominated by pale lager, extensive product standardization compressed sensory variability, limited the formation of distinctive flavor signatures, and weakened the emotional connection between brands and consumers. This convergence eroded brand loyalty and propelled the sector toward homogeneous competition [4,5]. Thus, breweries adopted flavor-centered differentiation strategies and sought to elevate overall product quality in order to maintain competitive advantage.

As the beer market shifted from an era of incremental growth to one of stock-based competition, firms increasingly relied on a precise understanding of consumers’ sensory preferences to secure differentiation in a highly homogeneous environment. Therefore, identifying how drinkers valued aroma, taste, and drinking sensation in pale lager constituted a critical foundation for product innovation and market planning. Descriptive and correlation analyses had been widely employed elsewhere to explore the links between consumer characteristics and product perception [6]. Relatedly, regression techniques offered a means to test causal mechanisms. Partial least-squares (PLS) regression, in particular, gained prominence in sensory and consumer research because it accommodated complex multivariate structures and yielded robust estimates even when data departed from normality [7,8]. Earlier work demonstrated that regression models could clarify how multiple factors jointly shaped consumer preferences, as shown in investigations of chilled meat-based ready meals, craft beer, and green-food repurchase intentions [9,10,11]. However, a comprehensive and quantitative assessment of these sensory priorities had yet to be undertaken.

Professional sensory evaluation had traditionally emphasized objectivity, reliability, and reproducibility, whereas consumer evaluation relied on simple questions such as “Do I like it?” and “Does it taste good?”. The latter more directly reflected market acceptance and commercial potential and was therefore regarded by scholars and practitioners as a necessary complement to expert-based methods. Earlier studies indicated that consumers generally assessed beverage quality holistically, while experts tended to dissect individual sensory elements [12]. For red wine, lay drinkers usually equated an overall smoother mouthfeel with higher quality, commonly expressed as lighter astringency, while professional assessors assigned greater importance to the interplay of several facets such as structural balance and complexity [13]. Comparable divergences were reported in beer evaluation, where tasting expertise shaped both assessment procedures and criteria [14,15]. Although these studies documented clear differences between consumer and expert judgements, most remained descriptive and offered few strategies for bridging the gap, leaving expert descriptors poorly aligned with actual drinking experience. The multicultural background and behavioral complexity of the Chinese market amplified this problem, as conventional evaluation frameworks seldom captured genuine consumer needs. Moreover, the food and beer industries are increasingly facing an urgent demand for faster and more cost-effective consumer testing methods [16,17]. To mitigate the discrepancies between consumer and expert evaluations and respond to this practical need, this study adopted a composite weighting approach that enabled the identification of key sensory attributes with a significant impact on consumer perception, while excluding those of minimal relevance. These prioritized attributes, together with their calculated weights, were then used to develop a novel sensory evaluation scale tailored to consumer preferences. Recent work in food studies applied the analytic hierarchy process (AHP) and the Entropy Weight Method (EWM) [18]. AHP derived subjective weightings through pairwise-comparison matrices, whereas EWM quantified the information content of each indicator by score dispersion; their combination reduced the subjectivity or data sensitivity inherent in single-model approaches and permitted a more comprehensive appraisal of attribute importance [19]. The technique of weights has been employed to quantify the relative influence of individual sensory attributes on overall quality perception [20,21].

In this study, a structured questionnaire targeting the pale lager market was designed and administered to obtain individual-level data. Descriptive and multivariate statistical procedures were applied to examine the purchase-decision structure across the four sensory dimensions (appearance, aroma, taste, and drinking sensation) to isolate the attributes exerting the greatest influence on choice and to establish which demographic factors independently shaped these sensory judgements. The AHP–EWM–Delphi method was subsequently integrated, from the consumer perspective, to quantify the relative importance of each sensory attribute in purchase intention and to establish a comprehensive weighting profile. Based on this, key sensory attributes were screened to reflect consumer priorities. A novel sensory evaluation scale was then constructed and further validated for its ability to align professional assessments with consumer preferences. This study aimed to fill a gap between sensory science and consumer-behavior research and to furnish breweries, especially those focused on pale lager, with actionable preference metrics and decision-support tools for product development, optimization, brand communication, and market segmentation.

## 2. Materials and Methods

### 2.1. Questionnaire Design

A multi-source information integration strategy was implemented to develop the questionnaire used to explore how sensory preferences affected purchase decisions. Relevant Chinese and international studies on beer consumption and sensory evaluation were first reviewed in a systematic manner, providing theoretical support for item generation and ensuring that each question aligned with established constructs. Next, content validity was examined through consultations with specialists in brewing, sensory science, and marketing; these experts discussed and scored every item, which led to revisions in terminology, dimensional coverage, and logical flow. Finally, a pilot survey was administered to a small, representative consumer group. Feedback on comprehension, wording clarity, and response time prompted further refinement, thereby improving the instrument’s reliability and response quality for large-scale data collection.

The final questionnaire comprised three sections: consumption habits, the perceived importance of sensory attributes in purchasing, and demographic background. The core section on sensory attributes centered on the four sensory dimensions—appearance, aroma, taste, and drinking sensation—and was further broken down into secondary and tertiary indicators to capture the full breadth of consumer attention to pale lager beer [22].

### 2.2. Data Collection

Data collection was conducted in China from 1 April to 1 June 2025 among respondents aged eighteen years and above. The questionnaire was administered online with the Questionnaire Star platform, and routine quality-control checks were applied to maintain completeness and representatives. In the first phase, 427 responses were gathered and used to assess feasibility and refine the instrument. The second phase yielded 1114 responses, which underwent preliminary structural inspection and logic-consistency testing to confirm data integrity at scale. During the final phase, 2085 questionnaires were distributed; after rigorous screening, 1837 valid cases were retained, producing an effective response rate of 88.11%. Two exclusion rules guided the screening process: individuals who reported “never drinking beer” were removed to ensure relevant tasting experience, and any record failing the embedded attention and logic checks was discarded. The resulting dataset satisfied the quantitative and structural requirements for subsequent empirical modeling and provided a sound basis for further analysis.

To assess the reliability and validity of the sensory rating scales before further statistical analysis, we applied several established evaluation procedures. The Kaiser–Meyer–Olkin (KMO) statistic, which measures sampling adequacy by evaluating the proportion of common variance among variables, was used to determine whether the dataset was suitable for structure detection—values above 0.70 are generally considered good. Bartlett’s test of sphericity, which tests the null hypothesis that the correlation matrix is an identity matrix, was applied to verify that sufficient intercorrelation existed among items for valid construct assessment, with *p* < 0.05 indicating suitability. Meeting these prerequisites, we conducted an exploratory factor analysis (EFA)—a statistical method used to identify the underlying factor structure of a set of observed variables—to verify that each item loaded appropriately on its intended construct; factor loadings above 0.60 and communalities exceeding 0.40 are regarded as acceptable indicators of construct validity. Internal consistency reliability was further assessed using Cronbach’s α, which quantifies the degree to which items within a scale measure the same underlying construct; coefficients above 0.70 are considered satisfactory [23,24,25]. Collectively, these procedures ensured that the selected multi-item scales demonstrated strong construct validity and measurement reliability, providing a sound foundation for subsequent correlation analyses and model estimation.

### 2.3. Descriptive and Correlation Analysis

Descriptive statistics were first computed to characterize the sample and to summarize score distributions for each sensory dimension. Spearman rank correlations were then calculated to reveal monotonic relations and preliminary trends among key variables, thereby providing theoretical support for subsequent modeling. Partial least-squares regression was applied to accommodate the multivariate structure in which several independent and dependent variables coexisted. Variable importance in projection (VIP) statistics were extracted to identify the demographic factors—sex, age, educational background, and spending willingness—that exerted the strongest independent influence on sensory evaluations [26,27].

### 2.4. Weight Index Analysis

Drinking frequency was selected as a grouping factor because it captured usage experience and involvement depth while maintaining perceptual consistency within each frequency band [28]. Respondents were therefore classified into three cohorts: 1–3 drinking occasions per month, 4–8 occasions, and 9 or more occasions. For each cohort, composite weights of the four sensory dimensions (appearance, aroma, taste, and drinking sensation) were estimated by means of the AHP and the EWM. Unlike traditional AHP, the present study derived subjective weights directly from consumer ratings. Because Likert scales encourage avoidance of extreme positions and may introduce central-tendency bias [29,30], mean scores for each attribute were first normalized by the min–max method and then linearly mapped onto the AHP one-to-nine scale [31,32]. To reduce the limitations of any single approach, the Delphi technique was employed to fuse subjective and objective weights. An expert panel anonymously reviewed the preliminary results, and a weighting ratio of seven to three was applied to combine AHP-based subjective weights with EWM-based objective weights, generating the composite indices, $W_{1,a}$, $W_{2,a}$, and $W_{3,a}$, for the three cohorts.

Comprehensive weights across cohorts were calculated by accounting for both within-group preferences and each group’s representativeness in the overall sample. A representative frequency value, $f_{i}$ (2, 6, and 14, respectively), and the corresponding sample size, $N_{i}$, were assigned to each cohort. A group contribution term, $M_{i}=f_{i}\times N_{i}$, was obtained, and normalized coefficients, $C_{i}=M_{i}/\sum M_{i}$, were computed such that $\sum C_{i}=1$. The overall weight for any sensory attribute, a, was then given by

$$W_{a}=C_{1}W_{1,a}+C_{2}W_{2,a}+C_{3}W_{3,a}$$

The sums of the $C_{i}$ and $W_{a}$ sets were checked and, if necessary, slightly normalized to ensure unit totals. The term $W_{k,a}$ represents the within-cohort weight assigned to sensory attribute a for cohort k. The final comprehensive weight, $W_{a}$, is obtained as the sum of the cohort-specific weights, each multiplied by the corresponding normalized contribution coefficient. This formulation accounts for both the importance of an attribute within each cohort and the cohort’s relative representativeness in the total population.

### 2.5. Sensory Evaluation Scale Construction

To ensure both scientific rigor and practical applicability, the construction of the sensory evaluation scale was organized into three sequential stages. In the first stage, sensory attributes with perceptual similarity were systematically consolidated based on principles of sensory cognitive psychology and consumer perceptual experience. This consolidation reduced redundancy and enhanced the conceptual coherence of the scale, in line with the “conceptual integration” approach [33,34]. Additionally, to maintain theoretical soundness and practical usability, the design process drew on the structure and item settings of existing sensory evaluation forms widely adopted by major beer companies and national standards (GB/T 4927-2008) [35]. Attributes showing high heterogeneity in consumer perception and limited explanatory value were excluded. In the second stage, attribute weights were determined using a “three-step standardization process,” which included local normalization, weighted integration, and global normalization. During local normalization, the top three sub-attributes within each primary sensory dimension were selected based on composite weight rankings, then proportionally scaled and standardized to ensure that their cumulative weight within each dimension totaled 100%. This step minimized the dilution of explanatory power from low-contribution indicators. In the weighted integration step, each standardized sub-attribute weight was multiplied by the corresponding primary dimension weight index to reflect the overall importance of each sub-attribute within the sensory structure. Finally, global normalization was applied to ensure that all sub-attribute weights summed to 100%, resulting in a mathematically closed and statistically coherent weighting system. In the final stage, the selected core attributes and their weights were used to develop a structured, consumer-oriented sensory evaluation scale for lager beer.

### 2.6. Sensory Evaluation Scale Validation

To evaluate whether the newly developed sensory evaluation scale for lager beer more accurately reflects consumer preferences, a two-group blind tasting experiment was designed. Rank correlation analyses were conducted to compare the consistency between the sample rankings derived from two sensory evaluation methods (the new scale and the existing scale) and consumer overall liking scores [36,37]. The objective was to determine whether the new scale could narrow the gap between professional assessments and actual consumer perceptions, thereby enhancing its applicability and market relevance.

The study included two groups of participants: a professional panel and a consumer panel. The professional panel comprised 10 sensory experts, each with at least two years of experience in beer sensory evaluation. The consumer panel consisted of 30 regular lager beer consumers aged 18–50, each consuming lager beer at least twice per month to ensure representative and diverse consumption habits.

Four representative lager beer samples (YJ, BW, HR, and QD) were selected from different brands, including both domestic and imported products, to provide sufficient sensory variation for validating the evaluation system. Specifically, YJ was a domestic beer with 2.5% ABV, BW was an imported beer with 3.5% ABV, HR was a domestic beer with 2.9% ABV, and QD was a domestic beer with 4.0% ABV. All samples were coded with three-digit random numbers to blind the participants to brand and origin information, ensuring the objectivity and reliability of the assessments. Within each session, the order of sample presentation was randomized individually for each assessor to avoid order effects. All beer samples were served at a controlled temperature of 2 ± 1 °C, which reflects typical consumer drinking conditions for pale lager. A standardized transparent tulip-shaped glass (150 mL) was used across all evaluations to ensure consistency in aroma and visual assessment. Sensory evaluations were conducted in a dedicated sensory evaluation room maintained at 25.0 ± 1.00 °C with relative humidity between 35.0% and 50.0%, in accordance with established sensory testing protocols. The environment was quiet, well-lit, and odor-free.

The professional panel conducted sensory evaluations of the same set of four lager beer samples using both scales. The existing sensory evaluation scale required panelists to assign an overall score based on a comprehensive descriptor, whereas the new scale asked them to score each core sensory attribute individually (1 = extremely weak, 9 = extremely strong). The overall scores for the new scale were then calculated using a weighted average of these attribute scores. To control for potential order effects, the evaluation sequence was randomized: 50% of the panelists used the existing scale first and then the new scale, while the remaining 50% followed the reverse sequence. A minimum interval of one day was maintained between the two sessions to reduce memory bias and sensory fatigue [38].

The consumer panel performed blind tastings of the same four samples. Each consumer provided an overall liking score on a 9-point hedonic scale (1 = dislike extremely, 9 = like extremely), reflecting their holistic sensory impression of each sample. Additionally, consumers were asked to rank the samples according to their preferences. These rankings were used to assess the concordance between consumer preferences and professional evaluations under both sensory scales.

### 2.7. Statistical Analysis

All statistical procedures were executed with Microsoft Excel 2019 and the online platform SPSSAU (https://spssau.com/, accessed on 11 June 2025). Graphs were prepared with Origin 2021 (OriginLab, Northampton, MA, USA) and the web-based tool ChiPlot (https://www.chiplot.online/, accessed on 2 July 2025).

## 3. Results

### 3.1. Questionnaire Optimization

The questionnaire underwent four systematic optimization rounds to secure scientific rigor, logical consistency, and practical applicability.

Round 1. A structural review was carried out jointly with a brewing enterprise and a market-research agency. Feedback from the Yanjing Brewery R&D team and Nielsen consultants indicated excessive length, highly technical wording, and overly closed response formats. The draft was therefore abridged, technical terms were converted into consumer-friendly expressions, and optional open fields were added for secondary and tertiary sensory items.

Round 2. A multidisciplinary focus group involving university students, corporate users, research-institute staff, and brewery employees evaluated wording and flow. The panel observed irregular sequencing, ambiguous wording in several items, poor recovery rates for open-ended questions, and misplaced privacy inquiries. In response, attention-check and contradiction items were inserted to enhance data authenticity, question order was reorganized from general to specific to improve navigability, and personal-information questions were moved to the end and rephrased to reduce respondent resistance.

Round 3. A pilot survey was conducted in which 100 questionnaires were distributed online at random and real-time feedback was collected. Results indicated that an overly long introduction prompted some participants to skip essential information, that non-drinkers were still required to complete all sections, and that certain technical terms and rating anchors lacked clarity. Consequently, the introduction was shortened and aligned more closely with the pale lager theme, skip patterns were programmed to target the intended population, a uniform five-point Likert scale was adopted to ensure comparability, and most open-ended questions were removed, leaving only essential structured fields.

Round 4. The fourth optimization round was driven by additional industry feedback. After reviewing earlier revisions, the research team and corporate partners confirmed pale lager as the exclusive focal product, refined screening questions, and expanded the list of lager-related flavor descriptors. These changes strengthened the topical focus and further improved the instrument’s validity.

After four rounds of refinement, the final questionnaire consisted of twenty-eight items organized into four sections: screening, consumption habits, perceived importance of sensory attributes for purchase decisions, and demographic information. Two attention-check items—a compliance task and a logic puzzle—were inserted to verify respondent attentiveness and to safeguard content validity. Five multi-item sensory rating scales were selected for reliability and validity analysis: Q11 (four sensory dimensions), Q12 (positive aromas), Q19 (negative aromas), Q20 (taste attributes), and Q22 (drinking sensations). Exploratory factor analysis showed factor loadings above 0.60 and communalities greater than 0.40 for every item. The cumulative variance explained by single-factor solutions exceeded 40 percent for each scale (Q11 = 56.49%, Q12 = 51.30%, Q19 = 44.79%, Q20 = 44.46%, Q22 = 46.54%). KMO statistics ranged from 0.765 to 0.947, and Bartlett’s tests were significant at *p* < 0.001, confirming suitability for factor analysis and satisfactory discriminant structure [39]. Internal consistency, assessed with Cronbach’s α, met or surpassed the 0.70 benchmark for all scales (Q11 = 0.742, Q12 = 0.810, Q19 = 0.823, Q20 = 0.750, Q22 = 0.895), with three scales exceeding 0.80, indicating good to excellent reliability [40]. Collectively, these results demonstrated strong reliability and construct validity, providing a sound measurement foundation for subsequent correlation analyses and model estimation.

### 3.2. Descriptive Analysis

#### 3.2.1. Background Information and Sample Composition

**Figure 1:**
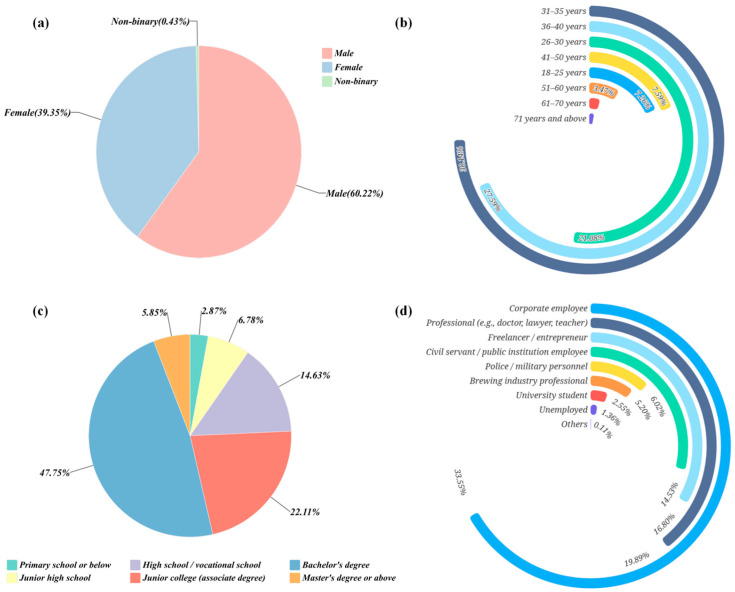
(**a**) Sex. (**b**) Age. (**c**) Educational background. (**d**) Occupational composition.

The demographic profile of the sample was judged to be both broad and structurally balanced (Figure 1a). With respect to sex, 60.22 percent of respondents were male and 39.35 percent were female, and 0.43 percent self-identified as non-binary. According to survey data, as of 2024, males account for nearly 60% of the beer-consuming population in China [41]. This distribution reflected prevailing consumption patterns in China’s beer market and ensured sufficient representation of all sexes, providing an appropriate basis for analyzing potential gender-related differences in sensory preferences.

The age range extended from eighteen to over seventy-one years, indicating wide coverage and inclusiveness (Figure 1b). Respondents aged twenty-six to forty years formed the principal cohort, accounting for 78.81 percent of the sample. The overall pattern approximated a normal distribution, an arrangement considered desirable in consumer-behavior research. The largest single group fell within the thirty-one to thirty-five bracket (30.14 percent), followed by those aged thirty-six to forty (27.59 percent) and twenty-six to thirty (21.08 percent). These groups typically occupied the peak of economic and social activity and thus exhibited relatively high purchasing power and brand engagement [42,43].

Educational background also displayed both breadth and hierarchy (Figure 1c). Respondents held qualifications ranging from primary school to postgraduate degrees. A total of 53.60 percent possessed at least a bachelor’s degree (bachelor’s degree: 47.75 percent; postgraduate and above: 5.85 percent), while 22.11 percent reported an associate degree and fewer than 10 percent had completed only secondary schooling or less. This distribution of educational background indicated a well-represented sample, thereby enhancing the credibility and depth of the sensory evaluation data.

Occupational composition further demonstrated breadth and variety (Figure 1d). Company employees formed the largest category (33.55 percent, *n* = 619). Professional and technical staff—including teachers, physicians, and lawyers—accounted for 19.89 percent, while freelancers or entrepreneurs represented 16.80 percent. Civil servants and public-sector employees made up 14.53 percent, and 5.20 percent of respondents worked directly in the brewing industry. This occupational composition underscored the comprehensive nature of the questionnaire and reinforced the reliability and representativeness of the data collected.

**Figure 2:**
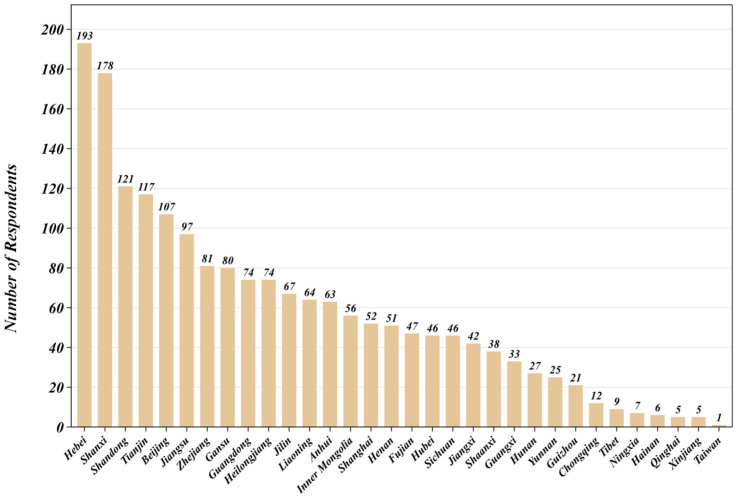
Regional distribution.

Regional distribution showed that the ten provinces or municipalities contributing the largest numbers of questionnaires were Hebei (193), Shanxi (178), Tianjin (117), Shandong (121), Beijing (107), Jiangsu (97), Gansu (80), Zhejiang (81), Heilongjiang (74), and Guangdong (74). The first six jurisdictions together supplied 813 cases, accounting for 44.13 percent of the valid sample, and thus indicated a pronounced spatial concentration (Figure 2). Most responses originated in the Bohai Economic Rim (Beijing–Tianjin–Hebei–Shandong–Shanxi) and the Yangtze River Delta (Shanghai–Jiangsu–Zhejiang), two established centers of beer production and consumption. Preferences observed in these areas were therefore expected to exert substantial influence on national demand patterns. Even so, questionnaires were also collected from provinces such as Shaanxi, Fujian, Liaoning, Hunan, Jiangxi, Sichuan, Henan, and Guangxi, ensuring coverage of cities at different administrative tiers and stages of economic development. The geographic profile thus balanced the purchasing power of coastal regions with the diversity of the country’s interior, providing a sound basis for cross-regional comparison [39].

Taken together, the sample displayed satisfactory balance across sex, age, educational background, region, and occupation. This breadth granted the study adequate population representativeness for analyzing consumer preferences, flavor choices, and sensory acceptance in China’s pale lager market.

#### 3.2.2. Consumption Behavior

**Figure 3:**
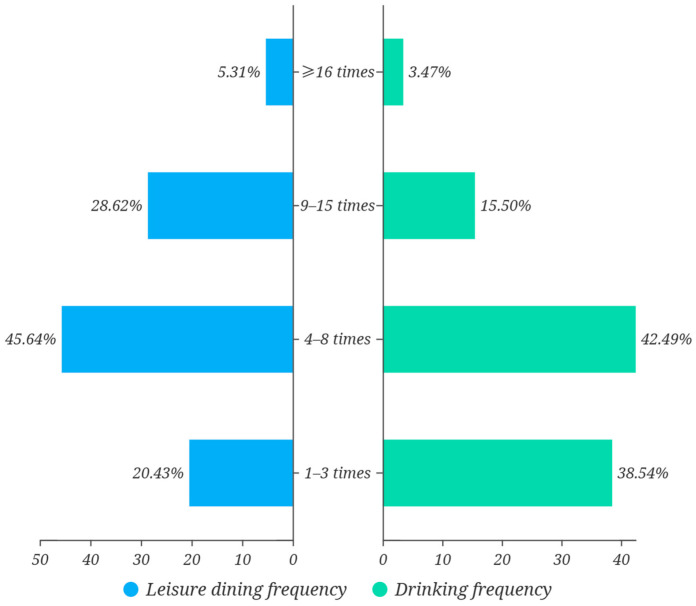
Leisure dining and drinking frequency.

**Figure 4:**
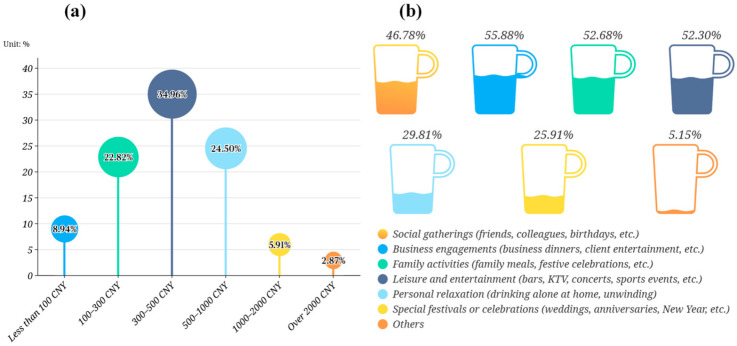
(**a**) Spending willingness distribution. (**b**) Drinking scenario distribution.

The survey indicated that respondents most frequently engaged in leisure dining at a moderate rate. Approximately 45.64 percent reported participating in such activities four to eight times per month, the most common interval (Figure 3). This pattern suggested a stable and regular habit of dining out, thereby providing ample occasions for the consumption of social beverages such as beer. Drinking-frequency data supported the characterization of a “moderate-consumption” profile. A total of 42.49 percent stated that they drank beer four to eight times each month, whereas 38.54 percent reported one to three occasions; thus, nearly 81 percent of the sample fell within the low-to-moderate range (Figure 3). This structure positioned the target market as a social, medium-frequency cohort rather than a group of heavy daily drinkers. In terms of spending willingness, 34.96% spent between CNY 300 and 500 (approximately USD 42.3–70.4) per month on alcoholic beverages, the most frequent bracket, and a further 22.82% fell between CNY 100 and 300 (approximately USD 14.1–42.3) (Figure 4a). These data implied relatively low spending per occasion and a preference for lower-strength, affordable products exemplified by brands such as pale lager beer brands. The behavioral pattern could therefore be summarized as “moderate frequency combined with budget control.” Scenario analysis confirmed the broad applicability of pale lager (Figure 4b). Social gatherings (46.78 percent), business entertainment (55.88 percent), family events (52.68 percent), and leisure activities (52.30 percent) all emerged as high-frequency contexts. Such situation-driven behavior indicated that beer consumption was deeply embedded in diverse social settings [44]. The versatility and light style of pale lager thus appeared well aligned with the needs of these varied occasions.

**Figure 5:**
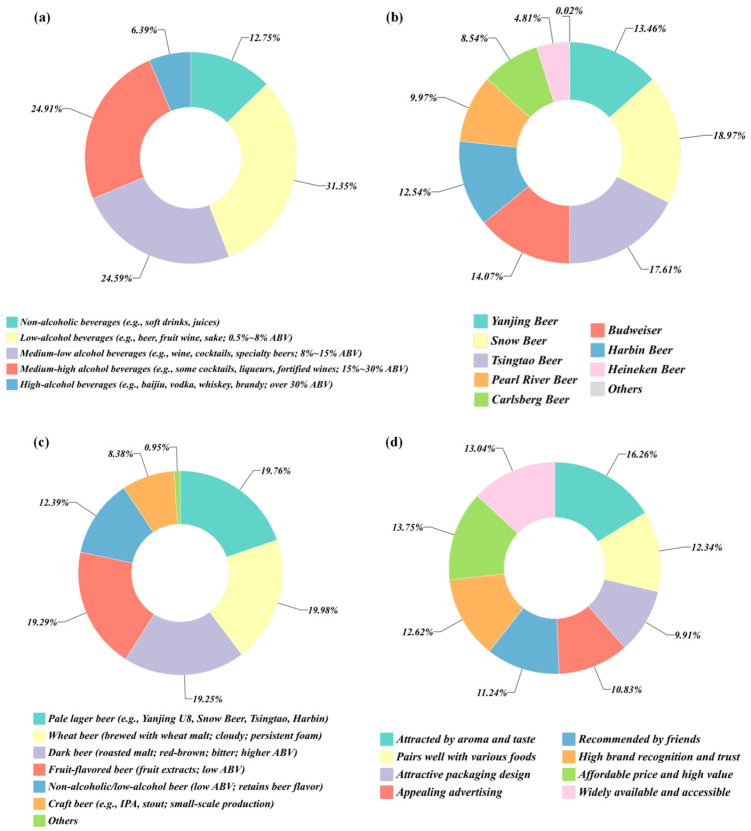
(**a**) Alcoholic strength preference. (**b**) Brand preference. (**c**) Preference for different beer. (**d**) Reasons for choosing.

Preference patterns for alcoholic strength are summarized in Figure 5a. A total of 68.62 percent of respondents indicated a preference for beverages containing 0.5–8 percent alcohol by volume, a range that corresponded closely to pale lager. This share exceeded the proportions favoring medium-strength drinks such as wine and cocktails (53.82 percent) and high-strength drinks such as Baijiu and whisky (13.98 percent), confirming that pale lager was accepted as an everyday option and that a general shift toward lower-alcohol, milder products had occurred [4,6]. Brand preferences are reported in Figure 5b. Snow (47.05 percent) and Tsingtao (43.69 percent) ranked highest, followed by Budweiser (34.91 percent) and Yanjing (33.39 percent). International labels such as Carlsberg (21.19 percent) and Heineken (11.92 percent) occupied smaller shares. The pattern implied strong familiarity with domestic brands, underscoring their dominant position in the pale lager segment, as Figure 5c shows [45]. Purchase drivers are presented in Figure 5d. Aroma and taste were identified as the primary considerations by 41.08 percent of respondents, while price (34.74 percent), channel convenience (32.95 percent), and brand awareness (31.87 percent) followed. The findings indicated a transition toward experience-oriented consumption in which intrinsic sensory quality outweighs traditional price concerns [46].

**Figure 6:**
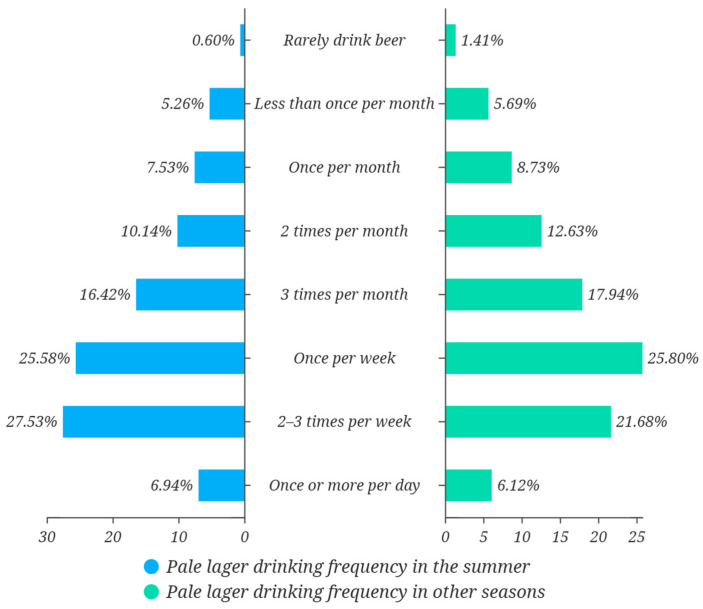
Pale lager drinking frequency in the summer and other seasons.

Seasonal variation in pale lager consumption was pronounced (Figure 6). During summer, 60.09 percent of respondents consumed the product at least once per week, and 27.53 percent reported a frequency of two to three times per week, confirming a marked surge in demand under high-temperature conditions. In the remaining months, the share of individuals who drank pale lager at least weekly declined only slightly to 53.62 percent, indicating that substantive usage persisted outside the peak season. Therefore, the data suggested that pale lager combined a strong seasonal uplift with stable off-season retention. It maintained a firm position in everyday drinking habits and enjoyed broad recognition as a mainstream beer style.

Overall, the respondents displayed a behavioral profile characterized by moderate drinking frequency, controlled expenditure, and sensory-driven preferences. Owing to its low alcohol concentration, mild flavor, and suitability for multiple occasions, pale lager matched the needs of the dominant consumer segment and remained both widely accepted and broadly consumed within the current beer market.

#### 3.2.3. Descriptive Findings on Sensory Preference

The evaluation scores revealed a clear multidimensional structure and nuanced preferences (Table A1). Mean ratings for all four sensory dimensions—appearance, aroma, taste, and drinking sensation—lay in the middle-to-upper range, indicating that consumers paid close attention to sensory cues when selecting pale lager [47,48]. Drinking sensation exerted the greatest influence; its mean score reached 3.737 (SD = 1.172), and 67.42 percent of respondents classified its impact as “high” or “very high,” underscoring the importance of mouthfeel attributes such as smoothness, coherence, and fullness. Appearance followed with a mean of 3.703 (SD = 1.070). Although slightly lower than the other dimensions, 46.94 percent of respondents assigned it a “high” impact and 21.46 percent a “very high” impact. Color and foam quality thus acted as an initial point of contact, guiding purchase judgements, particularly in visually oriented settings such as retail shelves and on-premise service, where a bright body and stable foam could quickly attract attention and generate a favorable first impression [49]. Mean scores for aroma and taste reached 3.696 (SD = 1.206) and 3.711 (SD = 1.143), respectively, confirming that olfactory and gustatory inputs formed the core of overall flavor perception [22]. These results collectively showed that consumers regarded both nose and palate as critical to product evaluation, while final acceptance was strongly conditioned by the integrated experience captured under drinking sensation.

The perceived influence of six representative aroma categories—malt, hop, fruit, floral, sweet, and fermentation—on purchase decisions for pale lager was assessed. Malt aroma emerged as the principal determinant. Its mean impact score reached 3.758 (SD = 1.136), and 68.62 percent of respondents rated this attribute as highly or very highly influential; the latter category alone accounted for 28.35 percent. Within the malt profile, grain aroma obtained the highest rating (M = 4.047, SD = 1.034), with more than three-quarters of participants indicating that they liked or strongly liked this note. Freshly baked bread (M = 3.872, SD = 1.078) and raw wheat (M = 3.835, SD = 1.002) also showed strong appeal. These findings suggested that the traditional grain-based character of a “malt-forward” style not only defined the classic flavor of pale lager but also guided actual purchase behavior whenever the product released natural grain and toasted cues [47]. Hop aroma recorded a mean score of 3.630 (SD = 1.155), lower than that of malt yet still in the upper-middle range; 24.12 percent classified its effect as moderate, and 25.20 percent as very high, indicating that nearly half of consumers adjusted their purchase decisions on the basis of hop notes [50]. Herbaceous freshness (M = 3.890, SD = 1.066) and lemon zest (M = 3.859, SD = 1.076) exerted the strongest positive influence, implying that clean, natural hop expressions most effectively stimulated buying interest. Mint (M = 3.853, SD = 1.073) and woody notes (M = 3.825, SD = 1.080) received steady support, whereas spicy-herb aromas (M = 3.783, SD = 1.120) ranked lowest and showed greater dispersion, suggesting that excessive pungency or complex spice profiles might dampen the expected “refreshing” perception and thereby curb purchase motivation [51]. Fruit aroma achieved a mean score of 3.629 (SD = 1.179), comparable to hop aroma; 19.78 percent reported a moderate effect and 24.77 percent a very high effect, indicating that more than forty percent modified their buying choices according to fruit notes [52]. Pineapple (M = 3.921, SD = 1.064) and apple (M = 3.915, SD = 1.029) led the rankings, confirming the strong consumer attraction to tropical sweetness and balanced sweet–acid flavor. Scores for grape (M = 3.864, SD = 1.078), citrus (M = 3.862, SD = 1.082), and banana (M = 3.826, SD = 1.123) were slightly lower yet remained positive, suggesting latent value for fruit aromas in youth-oriented product lines. Floral aroma produced a mean of 3.722 (SD = 1.169); 65.47 percent deemed its influence high or very high, reflecting good acceptance of jasmine, rose, or similar notes, possibly linked to current trends emphasizing natural fermentation and signature flavors. Sweet aroma averaged 3.713 (SD = 1.117), and 66.67 percent classified its impact as high or above. Honey (M = 3.878, SD = 1.063) and caramel (M = 3.858, SD = 1.045) received the strongest endorsement, while melon (M = 3.854, SD = 1.082) and roasted sweet potato (M = 3.825, SD = 1.131) also maintained favorable ratings, indicating a preference for mild sweetness. Fermentation aroma reached a mean of 3.738 (SD = 1.125); 65.64 percent considered it highly or very highly influential. Fresh-yeast-dough (M = 3.857, SD = 1.074) and light ethanol notes (M = 3.857, SD = 1.127) shared the top position, whereas sourdough (M = 3.814, SD = 1.120) and fermented-fruit acidity (M = 3.803, SD = 1.107) trailed slightly. Consumers therefore appeared receptive to the complexity imparted by natural fermentation but expressed lower tolerance for pronounced sour off-flavors.

The evaluation of off-flavor notes yielded an overall mean score of 3.767, indicating that such attributes exerted a noticeable negative effect on purchase decisions [53]. Phenolic odor, exemplified by disinfectant or medicinal aromas, recorded the highest aversion level (M = 3.854, SD = 1.095); 42.25 percent of respondents expressed dislike, and 29.54 percent reported extreme rejection, demonstrating a direct adverse impact on buying intent [54,55]. Fatty odor (M = 3.808, SD = 1.139), leather–tobacco notes (M = 3.812, SD = 1.095), oxidative odor (M = 3.752, SD = 1.147), sulfur compounds (M = 3.783, SD = 1.115), metallic odor (M = 3.772, SD = 1.133), and musty–earthy notes (M = 3.775, SD = 1.145) were also marked by many respondents as strong negative cues and were interpreted as signs of quality decline, thus markedly reducing purchase propensity. The green aldehydic odor, recognized as cucumber or grass notes, obtained the lowest mean within the set (M = 3.616, SD = 1.251), but its large standard deviation revealed substantial variability: some participants considered the note refreshing, whereas others regarded it as an immature off-flavor. Overall, the presence of off-flavor attributes—especially phenolic, oxidative, and fatty odors—was seen as highly detrimental to pale lager acceptance [56,57]. Breweries should strengthen post-fermentation filtration, employ oxygen-free packaging, and preserve cold-chain logistics, supported by combined sensory and chemical monitoring, to keep off-flavors below perception thresholds and to mitigate their negative impact on consumer purchase decisions.

The taste dimension comprised six basic flavors: sweetness, sourness, bitterness, saltiness, umami, and astringency. Among these, saltiness (M = 3.782, SD = 1.112) and astringency (M = 3.757, SD = 1.119) exerted the strongest influence on purchase decisions. A moderate mineral salinity or slight astringency was perceived by some consumers as enhancing complexity and reducing palate fatigue. Because saltiness is not a conventional target flavor in pale lager, its elevated impact required careful interpretation; if introduced by process or storage faults, the same note could have produced a negative effect. Scores for sweetness (M = 3.734, SD = 1.113), bitterness (M = 3.735, SD = 1.113), sourness (M = 3.711, SD = 1.126), and umami (M = 3.702, SD = 1.202) clustered between 3.702 and 3.735, indicating that consumers valued overall flavor balance rather than the high intensity of any single taste [58,59]. A harmonious sweet–bitter ratio conveyed the classic combination of malt richness and hop bitterness, moderate acidity refreshed the palate, and a trace of umami contributed to a rounded aftertaste.

Findings for the drinking sensation dimension further supported an experience-oriented purchasing pattern. Coordination (M = 3.770, SD = 1.228) and smoothness (M = 3.764, SD = 1.129) received the highest scores, suggesting that integrated flavor and easy swallowing encouraged inclusion in the purchase set. Perceived alcohol warmth (M = 3.741, SD = 1.121), spiciness (M = 3.742, SD = 1.133), and bloating sensation (M = 3.742, SD = 1.128) showed similar means, indicating that moderate strength, mild pungency, and low gastric fullness collectively defined pale lager’s “drinkability”; excessive intensity in any of these attributes would probably have reduced buying interest. Refreshing quality (M = 3.733, SD = 1.118) and aftertaste (M = 3.713, SD = 1.155) were also rated highly, underscoring the importance of a clean finish and pleasant lingering flavor. In contrast, prickliness (M = 3.690, SD = 1.156), body fullness (M = 3.720, SD = 1.106), viscosity (M = 3.726, SD = 1.151), and foam fineness (M = 3.688, SD = 1.141) exerted weaker effects, implying that these parameters merely needed to meet baseline expectations. In summary, smoothness and flavor coordination appeared to be the most decisive drivers of pale lager purchase.

### 3.3. Demographic Influences on Sensory Perception: Correlation and PLS Regression Analysis

In order to identify the independent effects of demographic factors (sex, age, educational background, and spending willingness) on beer sensory evaluations, Spearman correlation and partial least-squares regression (PLS) analyses were employed (Table A2 and Table A3).

Spearman correlation analysis initially examined the monotonic relationships between demographic factors and four major sensory dimensions—appearance, aroma, taste, and drinking sensation—and their specific attributes. Significant positive correlations were observed between educational background and spending willingness across all sensory dimensions (r = 0.127–0.199, *p* < 0.01). Age showed a moderate but consistent influence across these dimensions (r = 0.063–0.172, *p* < 0.05), whereas sex exhibited relatively weaker correlations (r = 0.046–0.092, *p* < 0.05). Specifically, appearance, encompassing attributes such as beer color, clarity, foam abundance, and foam fineness, showed notable positive correlations with educational background (r = 0.199, *p* < 0.01) and spending willingness (r = 0.196, *p* < 0.01). Older consumers also valued visual attributes slightly more (r = 0.097, *p* < 0.05), with sex presenting the weakest association (r = 0.092, *p* < 0.05). The aroma dimension, a critical indicator of beer flavor quality, revealed modest yet significant correlations with education (r = 0.165, *p* < 0.001) and spending willingness (r = 0.177, *p* < 0.001). Malt aroma specifically correlated positively with age, education, and spending willingness (r = 0.091–0.155, *p* < 0.001). Hop aroma showed similar associations, highlighting consumers’ sensitivity to specific flavor details linked to beer knowledge. Fruit and floral aromas appealed broadly, correlating significantly with all demographic variables, especially education (r = 0.056–0.183). Sweet aroma presented weaker correlations, mirroring the patterns observed for fruit and floral notes, while fermentation aroma strongly correlated with spending willingness (r = 0.175, *p* < 0.001). Taste evaluation, covering sweetness, sourness, bitterness, saltiness, umami, and astringency, showed that educational background significantly influenced perceptions of complex tastes (r = 0.165–0.185, *p* < 0.001). Bitterness, sourness, and umami attributes, in particular, were valued by educated consumers. Spending willingness also correlated positively with these complex taste attributes (r = 0.110–0.164, *p* < 0.001), with bitterness and astringency receiving notable attention. Sex exhibited minimal independent effects, primarily influencing sweetness perception slightly. Drinking sensation, encompassing attributes such as smoothness, fullness, aftertaste, alcohol warmth, prickliness, and bloating sensation, demonstrated that education strongly influenced evaluations, particularly regarding body coordination and perceived alcohol warmth (r = 0.124–0.215, *p* < 0.001). Spending willingness ranked second, with notable correlations observed for prickliness and bloating sensation (r = 0.163–0.167, *p* < 0.001), suggesting that high-budget consumers pay attention to carbonation levels and comfort after consumption. Age modestly correlated with fullness, aftertaste, and bloating sensations (r = 0.106–0.113, *p* < 0.05), while sex exhibited minimal correlations across attributes.

Subsequent partial least-squares regression analysis was conducted to isolate independent effects and clarify causal relationships among variables. Educational background emerged as the most robust predictor across all sensory dimensions (β = 0.175–0.239), especially influencing appearance details, comprehensive aroma evaluations, complex tastes, and sophisticated drinking sensations such as alcohol warmth and body coordination. Spending willingness consistently ranked second as an influential factor (β = 0.124–0.163, *p* < 0.001). It particularly impacted fermentation aroma purity, bitter–astringent taste complexity, and carbonation perception. Age demonstrated relatively modest independent influences (β = 0.066–0.15, *p* < 0.05), primarily affecting traditional tastes like bitterness and sourness, as well as aftertaste sensations. These findings imply that accumulated drinking experience heightens appreciation of complex flavors and subtle aftertaste characteristics. Sex, although significantly correlated in initial analyses, displayed minimal independent influence within the multivariate model (β ≈ 0.02–0.112, *p* < 0.05). Notably, sex only slightly affected sweetness perception, suggesting limited intrinsic gender differences once education and spending willingness were controlled for. Variable importance in projection (VIP) statistics further confirmed the hierarchy of these demographic influences. Educational background and spending willingness were identified as the most impactful predictors, with VIP scores of 1.44 and 1.13, respectively, considerably exceeding the scores for age (VIP = 0.61) and sex (VIP = 0.52). This ranking aligns closely with the regression outcomes, reinforcing that education and spending willingness significantly drive sensory judgments.

Overall, the integrated analysis highlights that beer consumers’ sensory preferences and evaluations are predominantly shaped by educational background and spending willingness, indicating that consumers possessing greater beer knowledge and higher purchasing power impose stricter sensory standards. Age contributes modestly, reflecting accumulated sensory experience and refined palate preferences. Sex shows minimal independent impact, particularly once educational and economic factors are controlled for. These insights provide valuable strategic direction for beer market segmentation, product development, and targeted consumer positioning strategies.

### 3.4. Weight Index Analysis for Groups with Different Drinking Frequencies

#### 3.4.1. Low-Frequency Drinkers (1–3 Occasions per Month)

**Figure 7:**
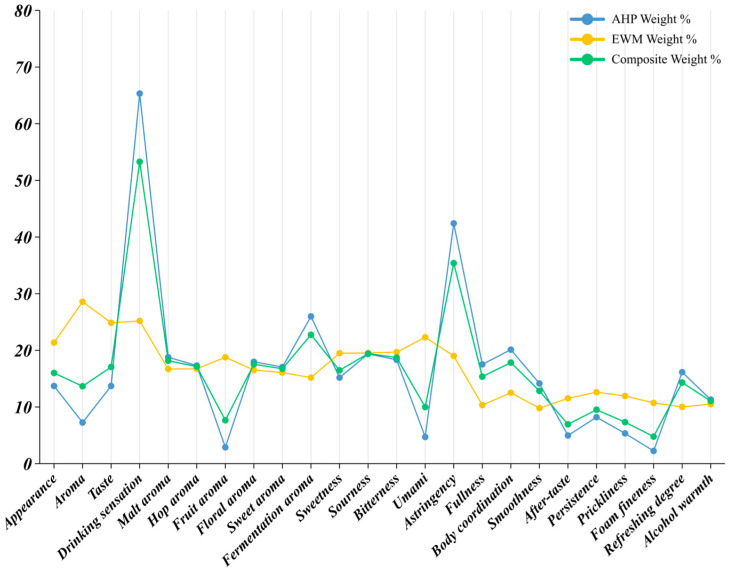
Weight index analysis of low-frequency drinkers (1–3 occasions per month).

The weighting structure for respondents who drank beer once to three times per month revealed their principal concerns and decision logic (Table A4 and Figure 7). Among the four sensory dimensions, drinking sensation dominated with a composite weight index of 53.28 percent, markedly surpassing the other three dimensions. AHP assigned this dimension 65.32 percent because self-ratings were consistently high, indicating that low-frequency drinkers placed greatest emphasis on ease of drinking and overall comfort rather than on detailed flavor cues. The EWM weight for drinking sensation reached 25.19 percent, although the ratings were relatively concentrated (high information entropy), and the standard deviation was smaller than for aroma or taste, implying strong consensus and limited discrimination. After combining AHP and EWM, drinking sensation remained the leading dimension, confirming its pivotal role. Taste (17.06 percent) and appearance (16.01 percent) ranked next. Clear color and persistent foam—a highly visible set of cues—retained moderate influence. Differences between the AHP and EWM values suggested that respondents perceived and judged these attributes but did so with little variation. Aroma came last at 13.66 percent. Despite an EWM weight of 28.58 percent, the AHP value reached only 7.26 percent, showing that low-frequency drinkers themselves gave aroma limited conscious weight, perhaps because aroma receives relatively little attention in everyday beer discourse. Within the aroma dimension, fermentation aroma had the highest composite weight (22.75 percent), mirroring its AHP share of 25.99 percent. Respondents thus regarded fresh-yeast notes as a fundamental indicator of beer quality and authenticity. Malt aroma (18.16 percent), floral aroma (17.54 percent), hop aroma (17.14 percent), and sweet aroma (16.75 percent) followed with fairly even weights, suggesting that, although aroma was not the primary driver, consumers could still discriminate between specific notes. Fruit aroma accounted for only 7.66 percent, indicating low sensitivity to ester-derived fruitiness, probably because this group rarely encountered craft beers with pronounced fruit profiles. In the taste dimension, astringency recorded the highest composite weight, 35.39 percent, the largest of all individual attributes. An AHP share of 42.41 percent demonstrated that respondents instinctively viewed astringency as a detractor from drinking pleasure. Even after adjustment for dispersion (EWM = 19.01 percent), the combined weight confirmed its negative dominance. Bitterness (18.73 percent) and sourness (19.42 percent) exerted medium-to-high influence: moderate bitterness may be accepted as a style marker, but excessive levels were penalized, whereas sourness attracted attention because it is uncommon in standard pale lager. Sweetness (16.47 percent) remained secondary, and umami (9.99 percent) exerted the weakest effect, likely because the amino acid complexity that generates umami is harder for general consumers to recognize or link to quality. Within drinking sensation, body coordination led with 17.84 percent, followed by fullness (15.36 percent), refreshment (14.31 percent), and smoothness (12.85 percent). These four positive attributes formed the core of the desirable experience, indicating a preference for harmonious, soft, naturally drinkable beer. Alcohol warmth (11.06 percent) and persistence (9.52 percent) occupied mid-level positions; while some respondents sought low alcohol intensity, most did not treat it as a decisive factor, and persistence drew limited attention because it is perceived only after consumption. Lower weights were assigned to prickliness (7.33 percent), aftertaste (6.94 percent), and foam fineness (4.78 percent), attributes closely aligned with professional sensory protocols and therefore less salient to ordinary buyers.

In summary, low-frequency drinkers focused first on overall drinking comfort, next on basic taste and visual cues, and least on aroma subtleties. Fermentation and malt aromas, moderate bitterness and sourness, and minimal astringency emerged as the principal flavor signposts guiding their purchase decisions.

#### 3.4.2. Mid-Frequency Drinkers (4–8 Occasions per Month)

**Figure 8:**
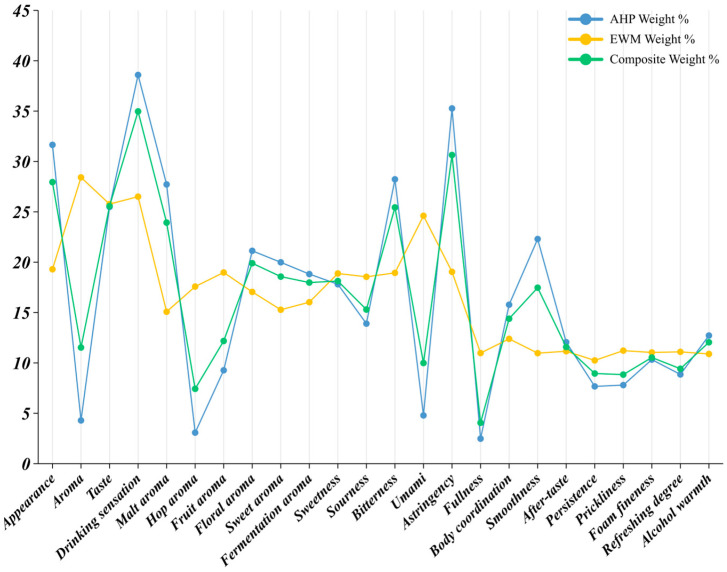
Weight index analysis of mid-frequency drinkers (4–8 occasions per month).

The weight index profile for respondents who drank pale lager four to eight times per month appeared more structured and diversified than that of the low-frequency cohort (Table A5 and Figure 8). Drinking sensation remained dominant, with a composite weight of 34.97 percent. The AHP share reached 38.59 percent and the EWM share 26.51 percent, indicating both strong subjective consensus and moderate score dispersion. The findings confirmed that this group paid close attention to overall mouthfeel—coordination, smoothness, and finish—when choosing a beer. Appearance followed at 27.95 percent and was the only dimension whose AHP weight exceeded thirty percent (31.65 percent), showing that visual cues were considered decisive at the point of purchase. The lower EWM figure (19.30 percent) suggested limited variation in the scores, meaning most respondents judged appearance in a similar way. Taste ranked third (25.57 percent). Its AHP and EWM values were almost identical (25.49 and 25.77 percent), confirming taste as a core decision factor and revealing meaningful inter-individual variation—evidence that respondents differed in the flavor aspects they valued. Aroma came last at 11.53 percent. A marked gap emerged between its AHP weight (4.29 percent) and its EWM weight (28.42 percent), implying that many respondents assigned aroma little conscious importance, yet a smaller subset gave it high scores, greatly increasing dispersion. The contrast suggested that recognizing aroma nuances required a higher expertise threshold than most mid-frequency drinkers possessed.

Within the aroma dimension, malt aroma led with a composite share of 23.93 percent; its high AHP figure (27.72 percent) indicated broad agreement that this note mattered strongly. Floral aroma (19.90 percent) and sweet aroma (18.57 percent) followed. Hop aroma displayed a pronounced subject–object gap: the AHP weight was only 3.08 percent, whereas the EWM weight reached 17.58 percent, giving a composite of 7.43 percent. This disparity showed that hop aroma divided the sample, likely reflecting differing exposure to more characterful beer styles. Bitterness (25.44 percent) and astringency (30.64 percent) had the greatest influence. Astringency’s exceptionally high AHP weight (35.27 percent) confirmed that any harsh mouth-drying effect was a major deterrent. Bitterness combined balanced AHP and EWM shares (28.23 and 18.94 percent), underscoring its dual role as a style marker and a potential negative cue at higher intensities. Sweetness (18.13 percent) and sourness (15.30 percent) exerted moderate influence. Umami recorded the lowest composite weight (9.99 percent) and a low AHP share (4.79 percent), but the high EWM share (24.61 percent) revealed wide scoring dispersion and limited average impact. Smoothness (17.47 percent) and body coordination (14.39 percent) topped the list, signaling that mid-frequency drinkers valued an integrated, easy-drinking texture. Aftertaste (11.58 percent), alcohol warmth (12.05 percent), and foam fineness (10.54 percent) occupied a middle tier. Persistence, prickliness, and refreshment ranged from 8.84 to 9.42 percent and played supporting roles. Fullness carried the lowest weight (4.06 percent), driven by a very small AHP share (2.48 percent); respondents therefore seldom treated fullness as a primary purchase criterion.

Mid-frequency consumers relied first on mouthfeel harmony, then on visual appeal and basic flavor, and only marginally on aroma. Within taste, they rewarded moderate bitterness and penalized astringency, whereas aroma judgements centered chiefly on malt and floral notes. These patterns suggested that product communication aimed at this segment should emphasize visual clarity, balanced bitterness, low astringency, and an overall smooth, coordinated drinking experience.

#### 3.4.3. Weight Index Profile of High-Frequency Drinkers (≥9 Drinking Occasions per Month)

**Figure 9:**
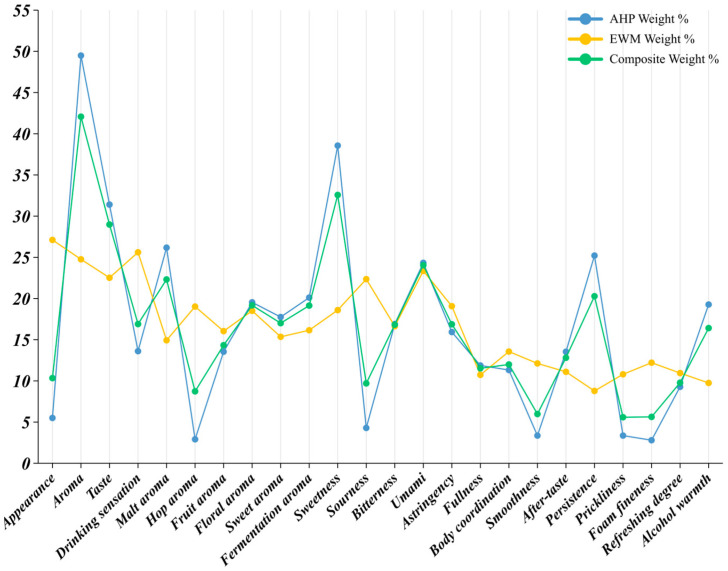
Weight index analysis of high-frequency drinkers (≥9 drinking occasions per month).

The weighting pattern for respondents who drank pale lager nine or more times per month differed markedly from those of the low- and mid-frequency groups (Table A6 and Figure 9). Aroma occupied an unequivocally dominant position, showing a composite weight of 42.07 percent. Its AHP share reached 49.49 percent, indicating that these consumers consciously regarded aroma as the most critical purchase criterion; the lower EWM share (24.76 percent) suggested relatively little score dispersion. Taste followed at 28.98 percent. The AHP (31.40 percent) and EWM (22.52 percent) values were close, implying both clear recognition and stable scoring. Frequent drinkers therefore maintained explicit, consistent judgements about flavor balance when selecting a beer. By contrast, appearance recorded an AHP weight of only 5.50 percent but an EWM weight of 27.11 percent, yielding a composite of 10.34 percent. Respondents thus stated that appearance mattered little, yet their scores varied widely, producing a high objective weight. Appearance consequently remained a secondary factor. Drinking sensation ranked third with a composite weight of 16.90 percent. Its modest AHP value (13.61 percent) showed low declared priority, whereas the higher EWM value (25.61 percent) reflected noticeable inter-individual variation. Hence, mouthfeel did not guide decisions for most high-frequency drinkers, but it elicited divergent personal reactions.

Within aroma, malt aroma led with a composite share of 22.31 percent. A low EWM value (14.93 percent) and a very high AHP value (26.17 percent) revealed broad agreement that malt notes were vital quality signals. Fermentation aroma (19.14 percent) and floral aroma (19.17 percent) followed, each displaying balanced AHP and EWM shares, indicating both high importance and good recognizability. Sweet aroma (17.01 percent) and fruit aroma (14.33 percent) were secondary. Hop aroma obtained only 8.73 percent overall: its AHP share was a mere 2.91 percent, whereas its EWM share rose to 19.02 percent, signaling pronounced disagreement. Sweetness produced the highest composite weight, 32.57 percent, and its AHP share reached 38.56 percent, far surpassing all other taste cues and confirming sweetness as the core quality indicator for frequent drinkers. Umami ranked second (24.04 percent) with closely matched AHP (24.33 percent) and EWM (23.35 percent) values, denoting consensus regarding its relevance. Bitterness (16.82 percent) and astringency (16.87 percent) carried moderate influence. Sourness had the lowest composite share (9.70 percent): the AHP value was only 4.29 percent, while the EWM value climbed to 22.35 percent, suggesting that, although most respondents downplayed acidity, a minority assigned it high scores. Persistence topped in the drinking sensation dimension at 20.28 percent; an AHP share of 25.21 percent revealed widespread concern for flavor longevity, whereas the low EWM share (8.78 percent) indicated minimal dispersion. Body coordination (11.99 percent) and fullness (11.53 percent) formed a middle tier. Alcohol warmth (16.41 percent; AHP 19.27 percent) ranked high, confirming that strong alcoholic perception constrained purchasing. Smoothness, refreshment, and aftertaste each exceeded five percent and served as secondary cues. Prickliness and foam fineness showed the smallest weights (5–6 percent), demonstrating that such physical details exerted limited impact on the choices of high-frequency drinkers.

High-frequency consumers relied primarily on aroma—especially malt, fermentation, and floral notes—followed by sweetness-centered taste cues. Appearance and most mouthfeel factors played minor or highly individual roles. Product strategies targeting this segment should therefore emphasize a distinct, high-quality aroma profile and maintain balanced sweetness and umami.

### 3.5. Comprehensive Weight Index Analysis

During the final aggregation step, frequency factors of 2, 6, and 14, determined through the Delphi method, were assigned to the low-frequency, medium-frequency, and high-frequency cohorts, respectively. When multiplied by the respective sample sizes, these factors produced population coefficients of 0.1285, 0.4269, and 0.4446. Each cohort’s composite weight for the four primary sensory dimensions was then averaged by these coefficients, yielding a market-wide comprehensive weight index.

**Figure 10:**
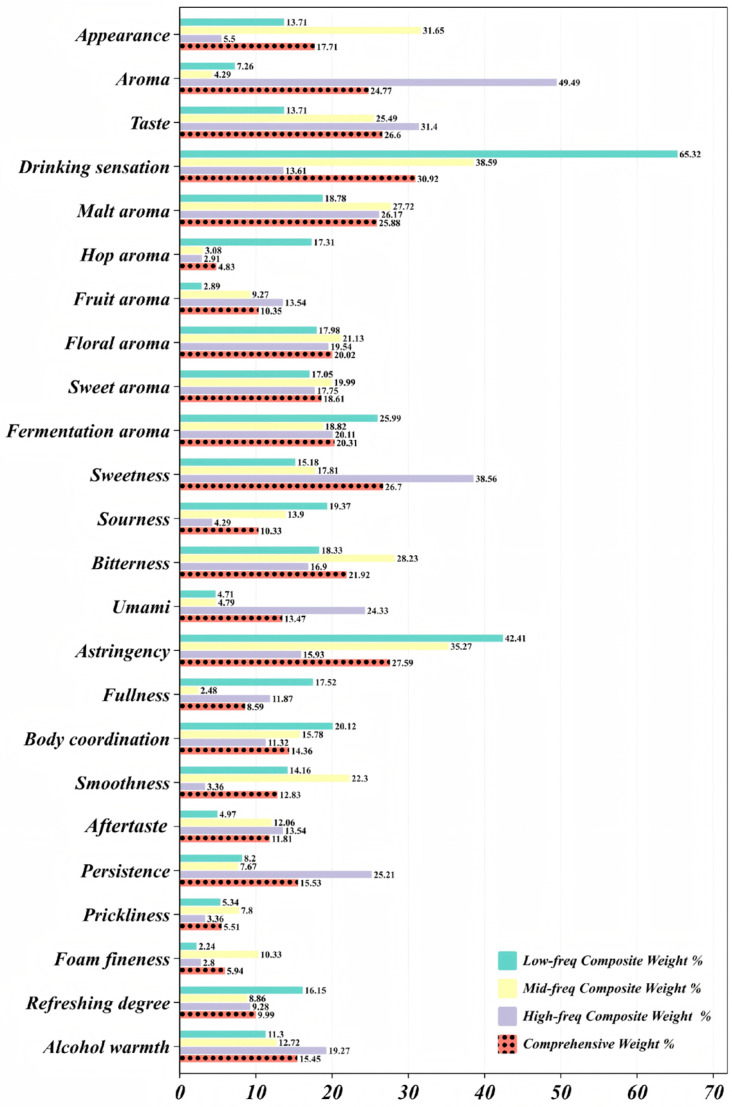
Composite and comprehensive weight index results.

The resulting distribution (Table A7 and Figure 10) indicated that drinking sensation remained the foremost driver of pale lager choice, with a comprehensive weight index of 30.92% [21]. Although high-frequency drinkers rated this dimension far lower (composite weight = 13.61%), the very large shares assigned by the low- and mid-frequency groups (65.32% and 38.59%), together with their balanced population coefficients, kept the aggregate index dominant. Taste followed at 26.60%. Cross-cohort variation was modest, yet high-frequency respondents still accorded it a conspicuous share (31.40%), confirming their sharper sensitivity to flavor layering. Aroma ranked third overall at 24.77%. Its low composite weights in the first two cohorts were offset by the high-frequency group’s very large weight (49.49%, population coefficient = 0.4446), showing that frequent drinkers relied increasingly on hop-, malt-, and yeast-derived notes when evaluating quality. Appearance carried the lowest comprehensive weight index, 17.71%. Mid-frequency drinkers considered it salient (31.65%), whereas high-frequency drinkers scarcely did (5.50%), implying that the influence of visual cues diminished as product experience accumulated.

Within the aroma dimension, malt aroma possessed the highest comprehensive weight index, 25.88%, emphasizing its universal role as a quality marker; its composite weight among high-frequency drinkers approached 26.17% [60]. Floral (20.02%) and fermentation aromas (20.31%) provided important secondary cues. By contrast, hop aroma showed the lowest index, 4.83%, because many consumers—particularly those with limited experience—found it difficult to identify reliably. Fruit (10.35%) and sweet aromas (18.61%) remained moderately influential; fruit notes appealed most to frequent drinkers.

For taste, astringency led with a comprehensive weight index of 27.59%, followed closely by sweetness (26.70%) and bitterness (21.92%). Together they accounted for more than three-quarters of the total taste weight, underscoring their structural dominance in flavor perception. Sourness (10.33%) and umami (13.47%) contributed less, often serving only as ancillary reference points; sourness was sometimes construed as a fault, whereas umami in pale lager was seldom recognized with precision.

Within drinking sensation, persistence (15.53%), alcohol warmth (15.45%), and body coordination (14.36%) formed the core of post-swallow evaluation. Persistence was weighted especially heavily by high-frequency drinkers, who relied on it to judge overall quality. Smoothness (12.83%) and aftertaste (11.81%) also retained notable influence. In contrast, fullness (8.59%), refreshment (9.99%), prickliness (5.51%), and foam fineness (5.94%) remained of limited consequence; foam cues, though classic brewing parameters, were perceived too variably by ordinary consumers to guide purchase decisions [61].

Collectively, the indices portrayed a flavor-led and experience-oriented preference structure. They highlighted the unequal importance of individual sensory attributes and the convergent tendencies that emerged across cohorts as flavor literacy grew [42]. The results offer clear guidance for mainstream product development: breweries should balance sweetness, astringency, and malt aroma while enhancing overall coordination and taste finesse to meet prevailing consumer expectations [62].

### 3.6. Sensory Evaluation Scale Construction

The construction of the sensory evaluation scale began with a refinement of the attribute framework to enhance clarity and applicability. Closely related sensory attributes were consolidated based on perceptual similarity to avoid redundancy and improve conceptual coherence. For example, “hop aroma” and “floral aroma” were combined into a single attribute due to their frequent co-recognition as plant-derived aromatic notes, while “fruity aroma” and “fermentation aroma” were integrated to represent a composite olfactory perception. Similarly, “aftertaste” and “persistence” were merged into “aftertaste–persistence” to better reflect the temporal aspects of post-consumption sensation [63,64].

To align the scale with both theoretical logic and industrial practicality, the attribute framework also referenced national standards and existing evaluation forms used by major beer producers. Attributes such as “alcohol sensation,” which are perceptible yet highly variable and difficult to quantify in consumer ratings, were excluded. This exclusion was justified on two grounds: first, alcohol sensation reflects physiological stimulation rather than hedonic quality; second, it is not considered an independent evaluative dimension in most current beer sensory standards. Removing such attributes improved the operational consistency and interpretability of the scale [64].

**Figure 11:**
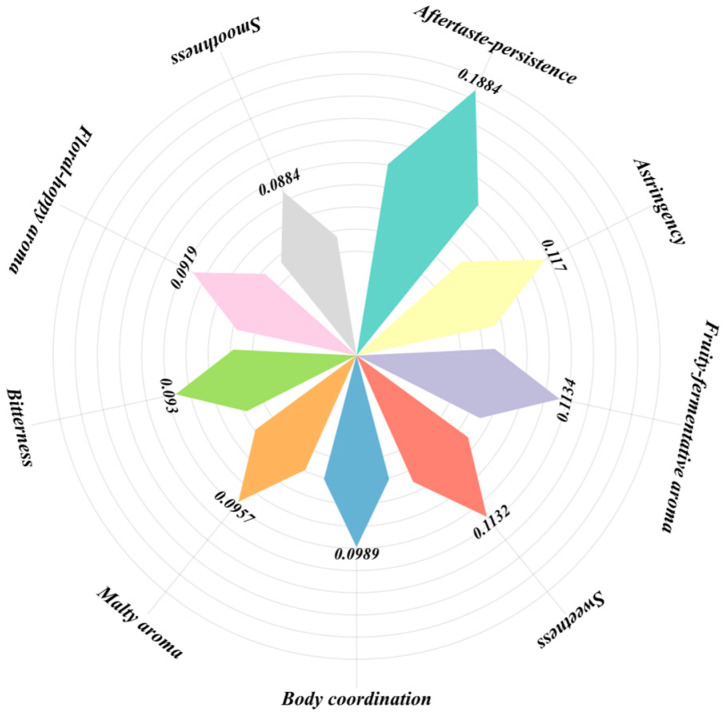
The final standardized weight results.

The resulting weighting structure highlighted consumer priorities in pale lager purchase decisions (Figure 11). “Aftertaste–persistence” emerged as the most influential attribute (18.84%), underscoring the importance consumers place on the lasting quality of the drinking experience. Within the taste dimension, “astringency” (11.70%) and “sweetness” (11.32%) ranked prominently, indicating consumer demand for structural complexity and palatability balance. In the aroma dimension, “fruity–fermentation aroma” (11.34%) demonstrated notable importance, revealing a preference for multidimensional and rich olfactory profiles. “Malt aroma” (9.57%) and “hop–flower aroma” (9.19%) also showed significant weights, confirming the central role of traditional ingredients and aromatic diversity in shaping consumer perceptions. Among drinking sensation attributes, “body coordination” (9.89%) reflected consumers’ emphasis on overall balance and smoothness. Although “bitterness” (9.30%) ranked slightly lower, it remained important for expressing beer style, and its moderate weighting suggests potential individual variability in preference. Lastly, “smoothness” (8.84%) maintained relevance, highlighting continued consumer attention to oral texture and drinkability.

Based on this analysis, nine sensory attributes with the highest influence on consumer perception were selected as the core framework for the new sensory evaluation scale. To facilitate practical application, the final standardized weights were converted into integer percentages summing to 100%.

### 3.7. Sensory Evaluation Scale Validation

To assess the predictive validity of the newly developed sensory evaluation scale relative to an existing industry-standard scale, rank correlation analyses were performed. The analysis compared the composite scores derived from both sensory evaluation methods with consumer preference rankings obtained through a nine-point hedonic scale. Two statistical metrics, Kendall’s tau (τ) and Spearman’s rho (ρ), were used as the primary indicators of concordance between professional evaluations and consumer preferences. This approach allowed for a rigorous assessment of each scale’s ability to reflect actual consumer sensory priorities.

**Figure 12:**
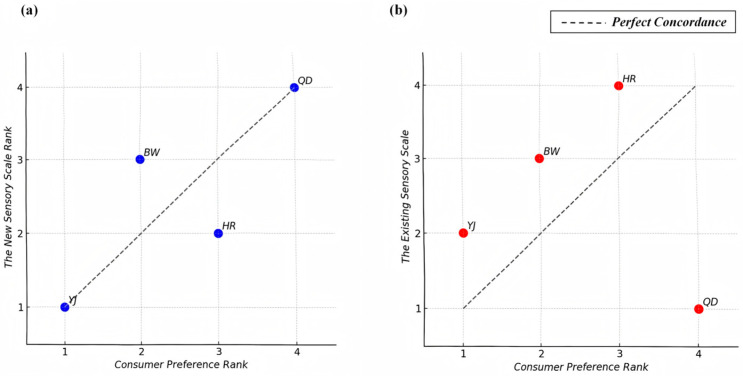
(**a**) Rank concordance between the new sensory evaluation scale and consumer preference rankings. (**b**) Rank concordance between the existing sensory evaluation scale and consumer preference rankings. The x-axis represents consumer preference rankings, and the y-axis represents rankings derived from each scale. The dashed diagonal lines indicate perfect concordance between professional and consumer rankings. Greater deviations from the diagonal reflect lower alignment.

The results demonstrated a substantial difference in alignment between the two sensory evaluation methods and consumer preferences (Figure 12a). The new scale yielded professional scores for YJ (6.61), HR (5.80), BW (5.75), and QD (3.98), corresponding to a ranking of YJ (1), HR (2), BW (3), and QD (4). Consumer preference scores for the same samples were YJ (7.43), BW (6.47), HR (5.97), and QD (4.83), resulting in a ranking of YJ (1), BW (2), HR (3), and QD (4). For the new scale, the ranking of samples showed strong concordance with the consumer preference ranking. The Kendall’s τ coefficient was 0.667, indicating a moderate-to-strong degree of ranking agreement, while the Spearman ρ coefficient reached 0.800, suggesting a similarly high level of monotonic relationship. These findings imply that the new scale can effectively capture the relative importance consumers assign to different sensory attributes and translate these perceptions into overall product evaluations that align closely with market preferences.

In contrast, the existing sensory scale produced professional scores for QD (6.40), YJ (6.20), BW (5.63), and HR (5.03), yielding a ranking of QD (1), YJ (2), BW (3), and HR (4) (Figure 12b). This ranking diverged substantially from the consumer preference ranking, with Kendall’s τ coefficient at 0.000 and Spearman’s ρ at −0.200. The negative Spearman correlation indicates an inverse relationship between the existing scale’s scores and consumer preferences, highlighting a misalignment in the evaluation of sample quality. This lack of concordance indicates that the current scale may not adequately represent the sensory drivers influencing consumer decisions in the lager beer category.

A further analysis of the sample rankings further highlights these discrepancies. The new scale’s composite scores ranked the four lager beer samples in nearly identical order to the consumer preference ranking, with only a minor inversion between the BW and HR samples. Specifically, the new scale placed HR slightly above BW, whereas consumers ranked BW marginally higher. Importantly, this deviation did not alter the overall trend and remained within an acceptable margin of variation. In sharp contrast, the existing sensory scale misclassified the samples in a way that conflicted with consumer preferences. Notably, it assigned the highest composite score to the QD sample, positioning it first in the ranking. However, consumer evaluations consistently placed QD at the bottom, suggesting a substantial divergence between the scale’s outcome and real-world consumer preference.

Collectively, these results support the validity of the newly developed sensory evaluation scale as a tool for bridging the gap between professional assessments and consumer perceptions. The high degree of ranking concordance observed for the new scale underscores its potential to provide breweries with actionable insights for product development, sensory optimization, and marketing strategies that are more closely aligned with consumer expectations.

## 4. Discussion

### 4.1. Demographic Influences on Sensory Perception

The present study provides valuable insights into how demographic factors shape consumers’ sensory perceptions and priorities when evaluating lager beer. Educational attainment consistently emerged as the most significant determinant of sensory evaluation, with standardized coefficients (β) ranging from 0.175 for appearance to 0.239 for drinking sensation. This pattern suggests that individuals with higher education levels exhibit heightened sensitivity to both the visual and gustatory qualities of beer. The strong associations between education and appearance attributes such as color, clarity, and foam quality (β = 0.175, *p* < 0.001) underscore the role of aesthetic awareness in shaping initial product impressions [65,66]. Similarly, educated consumers demonstrated pronounced responsiveness to aromatic complexity, as reflected by positive coefficients across malt (β = 0.179), hop (β = 0.159), fruit (β = 0.189), and floral (β = 0.192) notes. These results suggest that knowledge and exposure to diverse flavor experiences enhance the ability to discriminate subtle sensory nuances, aligning with findings from studies on wine consumers [67,68,69].

Spending willingness ranked second in influence, with significant positive effects across all sensory domains. High-budget consumers placed greater weight on fermentation aroma (β = 0.177), bitterness (β = 0.092), and astringency (β = 0.090), indicating a preference for robust and distinctive sensory signatures. Their heightened sensitivity to drinking sensations such as prickliness (β = 0.136) and bloating (β = 0.126) also highlights a concern for post-consumption comfort and carbonation balance, aspects increasingly emphasized in premium beer markets. These trends support previous findings that consumers willing to pay more often associate sensory richness and balance with product quality and value [70].

In contrast, sex and age demonstrated limited and largely attribute-specific contributions. The small positive coefficient for sex in sweetness preference (β ≈ 0.105) suggests a minor gender-related difference, while age showed weak associations with bitterness and sourness (β ≈ 0.10–0.15), possibly reflecting accumulated tasting experience rather than innate preferences. These minor effects imply that once education and spending willingness are accounted for, sex and age play secondary roles in determining sensory priorities. Such findings are consistent with earlier research showing minimal gender-based differences in beer evaluation when controlling for socioeconomic factors [71].

Although the R^2^ values for the models were modest (all below 0.10), the statistical significance in this large sample indicates stable demographic effects [72]. However, much of the remaining variance is likely attributable to individual differences in cultural background, physiological sensitivity, and prior exposure to diverse beer styles. These factors warrant further exploration using multi-level modeling or cross-cultural studies to better capture the complexity of consumer sensory perception.

Overall, these results suggest that breweries aiming to align their products with consumer expectations should pay particular attention to the preferences of highly educated and high-spending segments. These groups exhibit a greater appreciation for complexity in both flavor and mouthfeel, as well as heightened sensitivity to aesthetic and comfort-related aspects of beer consumption. Targeting these dimensions during product development and marketing could improve consumer satisfaction and competitive positioning in increasingly segmented beer markets.

### 4.2. Differential Sensory Attribute Preferences Across Drinking-Frequency Groups

The weighting results confirmed marked divergences among the three drinking-frequency cohorts. For low-frequency consumers, the composite index for drinking sensation reached 53.28 percent, far surpassing appearance (16.01 percent), taste (17.06 percent), and aroma (13.66 percent). This pattern indicated that novice drinkers relied primarily on holistic mouthfeel—smoothness, body coordination, and refreshment—when choosing pale lager. Limited flavor-recognition skills led them to judge beer chiefly by its ease of drinking and overall comfort. Their AHP weight for drinking sensation was very high (65.32 percent), while the corresponding EWM value was modest (25.19 percent), showing both strong subjective consensus and relatively little score dispersion. Among mid-frequency drinkers the sensory focus shifted. Although drinking sensation still ranked first (34.97 percent), its share fell markedly, whereas appearance rose to 27.95 percent and taste to 25.57 percent. Thus, once a moderate level of experience had been acquired, visual cues—color, clarity, and foam—became nearly as influential as basic flavor. Appearance received a high AHP weight (31.65 percent), reflecting a pronounced subjective judgement, and a lower EWM weight (19.30 percent), indicating some inter-individual variance. Aroma remained least important (11.53 percent), suggesting that odor appraisal had not yet become a primary decision factor. A different profile emerged for high-frequency drinkers. Aroma became the dominant dimension, with a composite weight of 42.07 percent, followed by taste at 28.98 percent. The AHP contribution for aroma reached 49.49 percent, confirming its critical role in conscious evaluation; the EWM value of 24.76 percent implied modest score dispersion and convergent preference. Drinking sensation dropped to 16.90 percent and appearance to 10.34 percent, showing that experienced drinkers depended less on immediate comfort or visual impression and more on discrete flavor cues when judging product quality. Collectively, the findings demonstrated a progression from an “overall comfort” orientation to a “structured flavor” orientation as drinking frequency increased. Occasional drinkers prioritized drinkability and visual immediacy, regular drinkers balanced mouthfeel with appearance and fundamental taste, frequent drinkers focused on nuanced aroma and taste attributes. These differences arose not only from varying levels of flavor-recognition proficiency but also from distinct expectations regarding the depth of the drinking experience.

Moreover, pronounced and finely grained differences were observed in the weighting of individual sensory attributes across the three drinking-frequency cohorts, reflecting both the stage of flavor-recognition maturity and the habitual expectations attached to the drinking experience [47]. Within the aroma dimension, malt aroma received a consistently high composite weight—18.16% for low-frequency, 23.93% for mid-frequency, and 22.31% for high-frequency drinkers—confirming its foundational role; the mid-frequency group relied on it most, suggesting that they still gauged quality mainly through basic, easily recognized aromas [73]. By contrast, the weight assigned to hop aroma, a more style-specific note, rose systematically from 4.83% to 7.43% and 8.73%, charting the progressive enhancement of sensory literacy [74]. Parallel tiered increases were recorded for floral and fruit aromas (floral: 17.54%, 19.90%, 19.17%; fruit: 7.66%, 12.18%, 14.33%), the latter derived chiefly from ester formation and therefore better appreciated by experienced drinkers who also preferred beers with fresher or tropical profiles [75]. Sweet aroma remained relatively stable (≈17–19%), indicating a broadly accepted hedonic role, whereas fermentation aroma carried the greatest weight among low-frequency drinkers (22.75%) and fell to 17.98% and 19.14% in the subsequent cohorts, suggesting that novices depended on strong, familiar yeast notes when judging typicality.

In the taste dimension, astringency ranked as the dominant deterrent for the first two cohorts, but its composite weight declined progressively (35.39% → 30.64% → 16.87%), showing that occasional drinkers equated astringency with physical discomfort, whereas frequent drinkers interpreted it as part of structural complexity [76]. Bitterness peaked in the mid-frequency group (25.44%), indicating that they had begun to value hop-driven character yet still reacted polarizingly. Meanwhile, high-frequency drinkers assigned markedly higher weights to sweetness (32.57%) and umami (24.04%), signifying a shift from isolated flavor cues toward overall palate harmony [77]. The influence of sourness diminished with experience (19.42% → 15.30% → 9.70%), consistent with the ability of seasoned drinkers to distinguish refreshing acidity from quality faults.

Within drinking sensation, low-frequency drinkers focused on body coordination (17.84%) and fullness (15.36%), revealing dependence on structural balance, whereas the mid-frequency cohort weighted smoothness (17.47%), aftertaste (11.58%), and alcohol warmth (12.05%), indicating a broader evaluation that embraced post-swallow attributes. High-frequency drinkers displayed the keenest interest in trailing sensations, assigning 20.28% to persistence and 12.81% to aftertaste, and they still attached 16.41% to alcohol warmth, reflecting refined tolerance thresholds and nuanced intensity judgements. Lower weights were given by this cohort to smoothness, prickliness, and foam fineness, implying a move away from surface cues toward depth of perception and flavor tension.

Overall, the differences in sub-dimensional weights among consumer groups with distinct drinking frequencies not only illuminated the sensory attributes emphasized when purchasing pale lager beer but also reflected the influence of demographic drivers, particularly the effects of educational background and spending willingness. Monthly alcohol budgets rose progressively with drinking frequency: in the low-frequency group, very few respondents spent more than CNY 500 per month, whereas in the high-frequency group more than 60 percent exceeded this threshold and almost one quarter allocated at least CNY 1000. As disposable income increased, consumers placed less weight on macro-level attributes such as basic smoothness and paid closer attention to micro-level features, including bitterness stratification and aftertaste complexity. Educational background followed the same developmental trajectory. Although low-frequency drinkers possessed a relatively dispersed educational profile, most reported an associate degree or bachelor’s degree. Mid-frequency drinkers were strongly concentrated at the bachelor’s degree level and shifted their focus from simple hedonic enjoyment to the suppression of negative stimuli such as astringency and off-flavors. Among high-frequency drinkers, the share holding a postgraduate degree more than doubled (11.46 percent compared with 5.24 percent in the low-frequency group and 3.96 percent in the medium-frequency group). This highly educated sub-group, equipped with more advanced tasting knowledge, sought greater aromatic precision and flavor balance. These findings indicated a staged professionalization of sensory cognition and product recognition strategies: consumers moved from reliance on overall mouthfeel, through heightened sensitivity to negative attributes, to an eventual focus on subtle hedonic details [78,79]. The observed associations between drinking frequency and demographic characteristics provide robust empirical evidence for tailoring flavor design, market segmentation, and communication strategies to specific target audiences.

### 4.3. Sensory Evaluation Scale Construction and Validation

The validation results of the newly developed sensory evaluation scale highlight its potential as a more effective tool for bridging the gap between professional assessments and consumer preferences in the lager beer category. Compared with the existing industry-standard scale, the new scale demonstrated substantially higher concordance with consumer preference rankings, as evidenced by its Kendall’s tau (τ = 0.667) and Spearman’s rho (ρ = 0.800). This marked improvement underscores a shift from expert-oriented evaluation frameworks toward more consumer-centered approaches.

One of the key features of the new scale lies in its integration of the Delphi method, entropy weight, and analytic hierarchy process (AHP), all applied from a consumer perspective and grounded in consumer survey data during its development. This multi-method strategy allowed for a balanced incorporation of consumer data, addressing a critical limitation of traditional sensory scales that often overlook market-driven factors. The current standard scale, which relies on a single overall descriptive score, offers operational simplicity but lacks fine-grained attribute weighting and fails to capture differential consumer sensitivities. In contrast, the new scale assigns higher weights to attributes such as “aftertaste–persistence” and “malt aroma,” reflecting contemporary consumers’ heightened attention to the persistence of drinking sensations and the complexity of aromatic profiles [80,81]. This adjustment enhances the scale’s explanatory power and provides product developers with actionable insights into flavor attributes most likely to influence purchasing decisions.

Furthermore, structural optimization of the attribute framework, including the consolidation of “hop aroma” and “floral aroma” as well as the exclusion of redundant items like “alcohol sensation,” aligns with principles of sensory cognitive psychology, such as perceptual coupling and semantic clustering [34]. These modifications reduce the cognitive load on assessors and improve scoring consistency, an important consideration given the divergent sensory representations and processing pathways between professional panelists and consumers. By minimizing ambiguity and enhancing perceptibility of individual attributes, the new scale narrows the semantic and cognitive gap between expert descriptors and consumer experiences, allowing professional assessments to more accurately mirror end-user perceptions.

From a practical standpoint, the new scale offers considerable advantages for brewery operations. Its strong alignment with consumer preferences suggests that it can serve as a robust tool for guiding product development, quality monitoring, and flavor communication in consumer education initiatives. As beer markets become increasingly fragmented and consumer expectations more diverse, the ability to quantify and prioritize attributes of greatest hedonic relevance becomes essential [73]. The data-driven weighting model embedded within the new scale ensures its adaptability across various stages of the product lifecycle, supporting not only sensory optimization during formulation but also consistent quality control in production and targeted marketing efforts. In contrast, the existing scale’s qualitative descriptors may lack the resolution needed for fine-tuning products to meet nuanced consumer demands, especially in the premium and craft segments.

In conclusion, the new sensory evaluation scale represents an advancement in aligning professional sensory analysis with consumer preferences. It maintains the rigor of traditional assessments while embedding consumer-driven weighting that reflects real-world drinking experiences. This approach constructs a consumer-oriented framework. The scale’s superior performance in predicting consumer preferences highlights its potential as a versatile tool for breweries seeking to innovate within an increasingly competitive and consumer-focused market landscape.

Although this study yielded valuable insights, several limitations should be recognized. First, the sample was drawn exclusively from China, which limits geographic and cultural diversity. Second, the questionnaire was distributed mainly through the Internet and social-media channels, so individuals who rarely use these platforms were probably under-represented, thereby reducing population coverage. Moreover, the validation was conducted using a limited sample set of four lager beer variants, which, although sufficient for initial testing, may not fully capture the broader variability present in different beer styles or within larger product portfolios. In addition, the consumer panel size was relatively small and may not fully represent the broader demographic diversity of lager beer consumers. Although participants were selected to include varying age groups and consumption habits, future studies should expand the sample size and scope for greater generalizability. Furthermore, while the professional panel consisted of trained participants, no formal calibration session was conducted prior to evaluation, which may have introduced minor variability in individual scoring. Implementing structured calibration procedures in future studies could further enhance panelist consistency and data reliability. To assess the scale’s generalizability, further research should include a wider range of beer categories and flavor profiles. Moreover, future studies could expand their scope through cross-regional or cross-cultural comparisons and supplement online recruitment with street-intercept or door-to-door surveys to enhance sample representativeness.

## 5. Conclusions

In this study, consumer sensory preferences were investigated in the pale lager beer category by integrating large-scale survey data with statistical modeling. The findings revealed that consumer preferences are shaped by multiple factors, with educational background and spending willingness being the most prominent. As consumption experience and spending willingness increased, consumer attention shifted from overall drinking sensation toward more specific sensory details such as stratified bitterness and aftertaste complexity. Educational background followed a similar pattern. These shifts reflected a professionalization in sensory perception, evolving from general preference to the suppression of negative stimuli and ultimately to the appreciation of fine sensory nuances. Drinking sensation, taste, and aroma were identified as the primary sensory drivers of pale lager choice, while appearance received the lowest weighting in the comprehensive weight index. Among these dimensions, malt aroma, astringency, and flavor persistence had the greatest influence on purchasing intent. Based on these results, a new sensory evaluation scale was designed to reflect consumer priorities more accurately. The scale focused on nine essential sensory attributes, each assigned a relative weight derived from combined analytic methods. Compared with the existing industry scale, the new approach demonstrated a stronger alignment with actual consumer rankings, confirming its practical effectiveness. The scale provides a simplified yet representative method for assessing pale lager products from the perspective of consumer expectations, with potential applications in product development, quality evaluation, and market positioning.

During the development of the scale, several key methodological considerations were also identified, particularly the challenge of balancing sample representativeness with research feasibility when integrating participants’ evaluations and large-scale consumer data. Based on these experiences, we recommend that future studies include calibration sessions prior to participants’ assessments and broaden recruitment channels to enhance demographic coverage. Moreover, the framework proposed in this study can be applied to other beer styles or beverage categories in different cultural and regional contexts, thereby further validating and extending the applicability and robustness of the scale.

## Data Availability

Data will be made available on request.

## Abbreviations

The following abbreviations are used in this manuscript:

AHP	Analytic Hierarchy Process
EWM	Entropy Weight Method

## Appendix A

**Table A1:** Descriptive statistics for sensory attribute ratings (*n* = 1837).

Sensory Attribute	Mean	SD
Appearance	3.703	1.07
Aroma	3.696	1.206
Taste	3.711	1.143
Drinking sensation	3.737	1.172
Malt aroma	3.758	1.136
Hop aroma	3.63	1.155
Fruit aroma	3.629	1.179
Floral aroma	3.722	1.169
Sweet aroma	3.713	1.117
Fermentation aroma	3.738	1.125
Wheat aroma	3.835	1.002
Cereal aroma	4.047	1.034
Fresh-bread aroma	3.872	1.078
Mint note	3.853	1.073
Spicy note	3.783	1.12
Lemon note	3.859	1.076
Woody note	3.825	1.08
Herbal note	3.89	1.066
Apple aroma	3.915	1.029
Banana aroma	3.826	1.123
Grape aroma	3.864	1.078
Pineapple aroma	3.921	1.064
Citrus aroma	3.862	1.082
Caramel note	3.858	1.045
Roasted-sweet-potato note	3.825	1.131
Honey note	3.878	1.063
Melon-sweet note	3.854	1.082
Fresh-yeast dough	3.857	1.074
Fermented-dough acidity	3.814	1.12
Fermented-fruit acidity	3.803	1.107
Light alcohol note	3.857	1.127
Phenolic odor	3.854	1.095
Aldehydic odor	3.616	1.251
Oxidative odor	3.752	1.147
Fatty odor	3.808	1.139
Sulphur odor	3.783	1.115
Metallic odor	3.772	1.133
Leather–tobacco odor	3.812	1.095
Musty–earthy odor	3.775	1.145
Sweet taste	3.734	1.113
Sour taste	3.711	1.126
Bitter taste	3.735	1.113
Salty taste	3.782	1.112
Umami taste	3.702	1.202
Astringency	3.757	1.119
Fullness	3.72	1.106
Body coordination	3.77	1.228
Smoothness	3.764	1.129
Aftertaste	3.713	1.155
Persistence	3.721	1.136
Richness	3.726	1.151
Prickliness	3.69	1.156
Foam fineness	3.688	1.141
Refreshment	3.733	1.118
Alcohol warmth	3.741	1.121
Spiciness	3.742	1.133
Bloating sensation	3.742	1.128

**Table A2:** Spearman correlation coefficients.

	Sex	Age	Educational Background	Spending Willingness
Appearance	0.082 ***	0.124 ***	0.145 ***	0.165 ***
Aroma	0.047 *	0.069 **	0.165 ***	0.177 ***
Taste	0.081 ***	0.063 **	0.165 ***	0.146 ***
Drinking sensation	0.073 **	0.081 ***	0.168 ***	0.130 ***
Malt aroma	0.027	0.091 ***	0.150 ***	0.155 ***
Hop aroma	0.028	0.090 ***	0.147 ***	0.108 ***
Fruit aroma	0.074 **	0.110 ***	0.176 ***	0.135 ***
Floral aroma	0.056 *	0.078 ***	0.183 ***	0.148 ***
Sweet aroma	0.046 *	0.111 ***	0.150 ***	0.152 ***
Fermentation aroma	0.083 ***	0.073 **	0.127 ***	0.175 ***
Sweetness	0.093 ***	0.110 ***	0.150 ***	0.120 ***
Sourness	−0.014	0.126 ***	0.184 ***	0.110 ***
Bitterness	−0.005	0.172 ***	0.171 ***	0.164 ***
Saltiness	0.008	0.128 ***	0.222 ***	0.137 ***
Umami	0.039	0.100 ***	0.185 ***	0.104 ***
Astringency	0.024	0.126 ***	0.183 ***	0.147 ***
Fullness	0.086 ***	0.110 ***	0.149 ***	0.114 ***
Body coordination	0.035	0.091 ***	0.178 ***	0.144 ***
Smoothness	0.089 ***	0.072 **	0.152 ***	0.124 ***
Aftertaste	0.072 **	0.106 ***	0.152 ***	0.145 ***
Persistence	0.050 *	0.079 ***	0.130 ***	0.110 ***
Richness	0.041	0.100 ***	0.133 ***	0.133 ***
Prickliness	0.034	0.101 ***	0.157 ***	0.163 ***
Foam fineness	0.053 *	0.099 ***	0.165 ***	0.142 ***
Refreshing degree	0.022	0.102 ***	0.124 ***	0.120 ***
Alcohol warmth	0.057 *	0.092 ***	0.215 ***	0.122 ***
Spiciness	0.008	0.075 **	0.173 ***	0.140 ***
Bloating sensation	0.032	0.113 ***	0.155 ***	0.167 ***

* *p* < 0.05, ** *p* < 0.01, *** *p* < 0.001.

**Table A3:** Regression coefficients (standardized β).

	Sex	Age	Educational Background	Spending Willingness
Appearance	0.112	0.066	0.175	0.148
Aroma	0.075	0.041	0.186	0.163
Taste	0.103	0.041	0.187	0.124
Drinking sensation	0.092	0.040	0.195	0.105
Malt aroma	0.052	0.061	0.179	0.133
Hop aroma	0.047	0.054	0.159	0.099
Fruit aroma	0.092	0.081	0.189	0.096
Floral aroma	0.092	0.027	0.192	0.125
Sweet aroma	0.067	0.081	0.186	0.121
Fermentation aroma	0.105	0.021	0.158	0.177
Sweetness	0.105	0.059	0.192	0.092
Fullness	0.113	0.075	0.181	0.086
Body coordination	0.069	0.061	0.201	0.103
Smoothness	0.110	0.032	0.193	0.095
Aftertaste	0.097	0.067	0.173	0.110
Persistence	0.075	0.064	0.172	0.082
Richness	0.072	0.066	0.160	0.093
Prickliness	0.039	0.070	0.173	0.136
Foam fineness	0.069	0.072	0.199	0.096
Refreshing degree	0.056	0.073	0.167	0.091
Alcohol warmth	0.091	0.077	0.239	0.079
Spiciness	0.033	0.039	0.198	0.106
Bloating sensation	0.053	0.076	0.195	0.126
Sourness	–0.005	0.095	0.222	0.057
Bitterness	–0.004	0.145	0.206	0.092
Saltiness	0.023	0.089	0.271	0.067
Umami	0.061	0.073	0.228	0.075
Astringency	0.048	0.089	0.228	0.090

**Table A4:** Weight index analysis of low-frequency drinkers (1–3 occasions per month).

Category	Sensory Dimension/Attribute	AHP Weight %	EWM Weight %	Composite Weight %
Primary dimensions	Appearance	13.71	21.37	16.01
	Aroma	7.26	28.58	13.66
	Taste	13.71	24.86	17.06
	Drinking sensation	65.32	25.19	53.28
Aroma attributes	Malt aroma	18.78	16.7	18.16
	Hop aroma	17.31	16.74	17.14
	Fruit aroma	2.89	18.78	7.66
	Floral aroma	17.98	16.52	17.54
	Sweet aroma	17.05	16.07	16.75
	Fermentation aroma	25.99	15.19	22.75
Taste attributes	Sweetness	15.18	19.49	16.47
	Sourness	19.37	19.53	19.42
	Bitterness	18.33	19.68	18.73
	Umami	4.71	22.3	9.99
	Astringency	42.41	19.01	35.39
Drinking sensation attributes	Fullness	17.52	10.34	15.36
	Body coordination	20.12	12.51	17.84
	Smoothness	14.16	9.8	12.85
	Aftertaste	4.97	11.53	6.94
	Persistence	8.2	12.61	9.52
	Prickliness	5.34	11.95	7.33
	Foam fineness	2.24	10.72	4.78
	Refreshing degree	16.15	10.02	14.31
	Alcohol warmth	11.3	10.51	11.06

**Table A5:** Weight index analysis of mid-frequency drinkers (4–8 occasions per month).

Category	Sensory Dimension/Attribute	AHP Weight %	EWM Weight %	Composite Weight %
Primary dimensions	Appearance	31.65	19.30	27.95
	Aroma	4.29	28.42	11.53
	Taste	25.49	25.77	25.57
	Drinking sensation	38.59	26.51	34.97
Aroma attributes	Malt aroma	27.72	15.08	23.93
	Hop aroma	3.08	17.58	7.43
	Fruit aroma	9.27	18.98	12.18
	Floral aroma	21.13	17.05	19.90
	Sweet aroma	19.99	15.28	18.57
	Fermentation aroma	18.82	16.03	17.98
Taste attributes	Sweetness	17.81	18.88	18.13
	Sourness	13.90	18.55	15.30
	Bitterness	28.23	18.94	25.44
	Umami	4.79	24.61	9.99
	Astringency	35.27	19.04	30.64
Drinking sensation attributes	Fullness	2.48	10.98	4.06
	Body coordination	15.78	12.39	14.39
	Smoothness	22.30	10.97	17.47
	Aftertaste	12.06	11.16	11.58
	Persistence	7.67	10.25	8.95
	Prickliness	7.80	11.22	8.84
	Foam fineness	10.33	11.04	10.54
	Refreshing degree	8.86	11.10	9.42
	Alcohol warmth	12.72	10.89	12.05

**Table A6:** Weight index analysis of high-frequency drinkers (≥9 drinking occasions per month).

Category	Sensory Dimension/Attribute	AHP Weight %	EWM Weight %	Composite Weight %
Primary dimensions	Appearance	5.50	27.11	10.34
	Aroma	49.49	24.76	42.07
	Taste	31.40	22.52	28.98
	Drinking sensation	13.61	25.61	16.90
Aroma attributes	Malt aroma	26.17	14.93	22.31
	Hop aroma	2.91	19.02	8.73
	Fruit aroma	13.54	16.04	14.33
	Floral aroma	19.54	18.51	19.17
	Sweet aroma	17.75	15.35	17.01
	Fermentation aroma	20.11	16.15	19.14
Taste attributes	Sweetness	38.56	18.59	32.57
	Sourness	4.29	22.35	9.70
	Bitterness	16.90	16.64	16.82
	Umami	24.33	23.35	24.04
	Astringency	15.93	19.07	16.87
Drinking sensation attributes	Fullness	11.87	10.73	11.53
	Body coordination	11.32	13.56	11.99
	Smoothness	3.36	12.12	5.98
	Aftertaste	13.54	11.10	12.81
	Persistence	25.21	8.78	20.28
	Prickliness	3.36	10.80	5.59
	Foam fineness	2.80	12.21	5.63
	Refreshing degree	9.28	10.95	9.78
	Alcohol warmth	19.27	9.75	16.41

**Table A7:** Comprehensive weight index results.

Category	Sensory Dimension/Attribute	Low-Freq. Composite Weight %	Mid-Freq. Composite Weight %	High-Freq. Composite Weight %	C_1_	C_2_	C_3_	Comprehensive Weight %
Dimension	Appearance	13.71	31.65	5.5	0.1285	0.4269	0.4446	17.71
	Aroma	7.26	4.29	49.49	0.1285	0.4269	0.4446	24.77
	Taste	13.71	25.49	31.4	0.1285	0.4269	0.4446	26.6
	Drinking sensation	65.32	38.59	13.61	0.1285	0.4269	0.4446	30.92
Aroma attributes	Malt aroma	18.78	27.72	26.17	0.1285	0.4269	0.4446	25.88
	Hop aroma	17.31	3.08	2.91	0.1285	0.4269	0.4446	4.83
	Fruit aroma	2.89	9.27	13.54	0.1285	0.4269	0.4446	10.35
	Floral aroma	17.98	21.13	19.54	0.1285	0.4269	0.4446	20.02
	Sweet aroma	17.05	19.99	17.75	0.1285	0.4269	0.4446	18.61
	Fermentation aroma	25.99	18.82	20.11	0.1285	0.4269	0.4446	20.31
Taste attributes	Sweetness	15.18	17.81	38.56	0.1285	0.4269	0.4446	26.7
	Sourness	19.37	13.9	4.29	0.1285	0.4269	0.4446	10.33
	Bitterness	18.33	28.23	16.9	0.1285	0.4269	0.4446	21.92
	Umami	4.71	4.79	24.33	0.1285	0.4269	0.4446	13.47
	Astringency	42.41	35.27	15.93	0.1285	0.4269	0.4446	27.59
Drinking sensation attributes	Fullness	17.52	2.48	11.87	0.1285	0.4269	0.4446	8.59
	Body coordination	20.12	15.78	11.32	0.1285	0.4269	0.4446	14.36
	Smoothness	14.16	22.3	3.36	0.1285	0.4269	0.4446	12.83
	Aftertaste	4.97	12.06	13.54	0.1285	0.4269	0.4446	11.81
	Persistence	8.2	7.67	25.21	0.1285	0.4269	0.4446	15.53
	Prickliness	5.34	7.8	3.36	0.1285	0.4269	0.4446	5.51
	Foam fineness	2.24	10.33	2.8	0.1285	0.4269	0.4446	5.94
	Refreshing degree	16.15	8.86	9.28	0.1285	0.4269	0.4446	9.99
	Alcohol warmth	11.3	12.72	19.27	0.1285	0.4269	0.4446	15.45
